# Experiences and Preferences in Zambia and South Africa for Delivery of HIV Treatment During a Client’s First Six Months: Results of the PREFER Study’s Cross-Sectional Baseline Survey

**DOI:** 10.1007/s10461-025-04640-y

**Published:** 2025-02-01

**Authors:** Nyasha Mutanda, Allison Morgan, Aniset Kamanga, Linda Sande, Vinolia Ntjikelane, Mhairi Maskew, Prudence Haimbe, Priscilla Lumano-Mulenga, Sydney Rosen, Nancy Scott

**Affiliations:** 1 Health Economics and Epidemiology Research Office, Faculty of Health Sciences, University of the Witwatersrand, Johannesburg, South Africa; 2 Department of Global Health, Boston University School of Public Health, Boston, USA; 3 Clinton Health Access Initiative-Zambia, Lusaka, Zambia; 4 Ministry of Health, Lusaka, Zambia

**Keywords:** HIV, Antiretroviral treatment, Early treatment period, Client-centered care, Preferences, South Africa, Zambia

## Abstract

Disengagement from antiretroviral therapy (ART) is highest in the early treatment period (≤ 6 months after initiation/re-initiation), but low intensity models designed to increase retention generally exclude these clients. We describe client preferences for HIV service delivery in the early treatment period. From 9/2022 to 6/2023, we surveyed adult clients who were initiating or on ART for ≤ 6 months at primary health facilities in South Africa and Zambia. We collected data on experiences with and preferences for HIV treatment. We enrolled 1,098 participants in South Africa (72% female, median age 33) and 771 in Zambia (67% female, median age 32), 38% and 34% of whom were initiating/re-initiating ART in each country, respectively. While clients expressed varied preferences, most participants (94% in South Africa, 87% in Zambia) were not offered choices regarding service delivery. 82% of participants in South Africa and 36% in Zambia reported receiving a 1-month supply of medication at their most recent visit; however, South African participants preferred 2- or 3-month dispensing (69%), while Zambian participants preferred 3-or 6-month dispensing (85%). Many South African participants (65%) would prefer to collect medication in community settings, while Zambian participants (70%) preferred clinic-based collection. Half of participants desired more one-on-one counselling and health information. Most participants reported positive experiences with providers, but long waiting queues were reported by South African participants.During the first six months on ART, many clients would prefer less frequent clinic visits, longer dispensing intervals, and frequent, high-quality counselling. Care models for the early treatment period should reflect these preferences.

*Registration*: Clinicaltrials.gov NCT05454839, Clinicaltrials.gov NCT05454852.

**Supplementary Information:**

The online version contains supplementary material available at 10.1007/s10461-025-04640-y.

## Introduction

HIV prevalence and incidence remain unacceptably high in many countries in sub-Saharan Africa despite two decades of large-scale HIV treatment availability. According to the most recent estimates from UNAIDS [1], South Africa, the country with the world's most severe HIV epidemic, had 7.7 million people living with HIV (PWH) in 2023, reflecting an adult HIV prevalence of 17.1%, while Zambia, another highly affected country, had 1.3 million PWH and an adult prevalence of 9.8%. Coverage with antiretroviral therapy (ART) was estimated at 77% and 95% of PWH in South Africa and Zambia, respectively. South Africa faces a large gap between estimated treatment coverage and the World Health Organization's global target of treating 95% of those who know their HIV status [2]. While overall treatment coverage in Zambia is high, both countries struggle with high rates of treatment interruption and disengagement which are generally not reflected in national treatment coverage estimates. Recent estimates of 12-month retention in care among those initiating ART between 2018 and 2021 were 61% in South Africa [3] and 46% in Zambia [4], including clients who interrupted treatment but returned to care by 12 months. These high rates of disengagement threaten achievement of national and global goals and are the focus of a large proportion of current attention [5].

Among HIV treatment clients across sub-Saharan Africa, the first six months after treatment initiation or re-initiation poses the greatest risk of disengagement from care (stopping treatment). Dubbed the “early treatment period” [6], this interval accounts for roughly three quarters of all first-year attrition from antiretroviral treatment (ART) [7]. In Zambia in 2018–2021, for example, more than a third of clients (34%) experienced interruptions to treatment (ITT) of 28 days or more during their first three months; another 16% interrupted treatment in months 4–6 [4, 8]. By months 7–12, the proportion of clients experiencing ITT had dropped to 12%, after which it plateaued to about 10% per year. During the same years in South Africa, only 56% of clients remained continuously on ART, without any interruptions > 28 days, in their first six months after initiation [3].

One challenge to increasing continuity of care during the early treatment period is that newly initiating or re-initiating clients are not eligible for most of the user-friendly differentiated service delivery (DSD) models that have been developed in recent years. DSD models such as six-month dispensing and adherence groups are client-centered approaches that reduce the burden of clinic visits, bring services closer to clients’ homes, and offer some degree of individual choice in determining how services will be accessed [9]. Eligibility criteria for these “low intensity” models of care usually include at least six months of treatment experience and documentation of a suppressed viral load. During the early treatment period, before these criteria can be met, clients are generally required to make frequent clinic visits and receive shorter-duration medication refills. COVID-19 restrictions precipitated some reductions in required numbers of clinic visits and increases in dispensing intervals [10], and new guidelines in South Africa now allow enrollment in DSD models after 4 months on ART, rather than 6 months [11], but for most clients, the early treatment period remains relatively burdensome and inflexible [12].

Adapting DSD strategies to meet clients’ personal preferences during the early treatment period has been proposed as one solution to high early attrition from care [13]. There is limited evidence that offering DSD enrollment in the early treatment period is feasible and successful [14], but since few if any countries have attempted it at scale, outcomes, challenges, and benefits are not well documented. One step in considering extension of DSD to the early treatment period is to understand patient preferences during this period. While a number of studies have described preferences for service delivery over the long term, after DSD eligibility [15–20], we are not aware of previous examinations of preferences during the early treatment period, which may differ from later years. Here, we report results from a study called PREFER (Preferences for services in a patient's first six months on antiretroviral therapy for HIV in South Africa and Zambia) [21]). PREFER's goal was to describe preferences for and experiences of HIV care and treatment among ART clients in the early treatment period in South Africa and Zambia in order to inform the design of DSD models for the early HIV treatment period and potentially improve early treatment outcomes.

## Methods

PREFER [21] was a mixed-methods, multi-component, prospective study of adult ART clients who were either initiating or reinitiating ART or had initiated ART within the previous 6 months at primary healthcare facilities in South Africa and Zambia. The study included a baseline survey, focus group discussions, and medical record follow up. The baseline survey addressed new clients’ characteristics, HIV care histories and experiences, resources, needs, concerns, and preferences to help inform the development of appropriate DSD models for the early treatment period. This paper uses the quantitative data from the baseline survey to report on prior experience with HIV care (Domain IV of the survey instrument) and self-reported preferences for how HIV treatment should be delivered during the early treatment period (Domain VI), two of the survey's core research questions, in both countries (see Supplementary file S1 for survey instrument). Other PREFER baseline survey results and other components of the PREFER study will be reported separately.

At the time of the study, both South Africa and Zambia had scaled up differentiated service delivery models nationally. In South Africa, widely available differentiated models of care (DMOC) included facility-based pickup points, external (community-based) pickup points, and adherence clubs. Although adherence clubs were originally popular, at the time of study enrolment, group models had been scaled back significantly due to COVID-19, and we did not encounter adherence clubs at most of the study sites. Facility-based and external pickup points allow established ART clients to pick up medications at convenient locations without waiting in long clinic queues. They generally required only two full clinical consultations per year, along with three or four medication pickups. In Zambia, the most common active model of care during the study period was facility-based six-month dispensing of antiretroviral medications, which requires only two healthcare system interactions per year. Other models included community ART access points, scholar models, and adherence clubs. For both countries, descriptions of DSD models have been widely published and specified in national guidelines [9, 22–25]. Early treatment period clients were not eligible for any of these models at the time the PREFER baseline survey was conducted.

### Study Sites and Population

PREFER was conducted at 12 public, primary healthcare facilities in Zambia and 18 in South Africa. They were selected to provide diversity in location (district and province), setting (rural, urban), client volume, DSD model offerings, and nongovernmental support partners. Further descriptions of the study sites have been reported previously [21].

PREFER enrolled new and recent ART initiators who were not yet eligible for low-intensity DSD models. In both countries, eligibility criteria for DSD enrollment at the time of the PREFER survey included i) at least six months on ART; ii) a documented suppressed viral load at the most recent test; and iii) no uncontrolled chronic conditions or co-morbidities, such as noncommunicable diseases, or opportunistic infections that may otherwise compromise clients’ health [26–28]. Inclusion criteria for PREFER study enrollment included living with HIV, being ≥ 18 years of age, and initiating or re-initiating ART on the day of study enrollment or within the six-month period preceding study enrollment.

As PREFER was a descriptive study that aimed to document participants’ self-reported experiences and preferences, rather than comparing outcomes or testing a hypothesis, the sample size for each country was chosen to optimize the use of study resources and time availability. Using the expected number of study-eligible patients at each study site, the number of such patients who were expected to visit the sites during the data collection period, and an anticipated data collection period of 90 days in each country, our minimum target was an average of 50 participants per study site, or 900 in South Africa and 600 in Zambia, with a maximum permitted by the ethics protocol of 2,500 per country. Based on our own prior experience, we anticipated that our minimum sample sizes to be sufficient to generate sufficiently precise results for the survey’s quantitative questions.

### Recruitment and Data Collection

Upon arrival at study clinics, clients seeking scheduled or unscheduled HIV treatment services were referred to a study research assistant by clinic staff. The research assistant screened potential participants for eligibility and, for those eligible, conducted the informed consent process. Clients who provided written informed consent were enrolled in the study and administered the baseline survey. The questionnaire contained eight thematic sections, including participants’ demographic characteristics and socio-economic status, HIV treatment history, current HIV care and treatment experience, and preferences for features of treatment delivery. Questions on preferences included visit time and frequency, provider interactions, service location, medication dispensing, counselling and health education. On the assumption that an individual’s prior experience with HIV testing and treatment is an important factor in the outcomes of the early treatment period, PREFER also asked several questions about HIV care history. Questions about clients’ perceptions of clinical care were answered using a 5-point Likert scale, describing the proportion of participants who agreed, disagreed, or neither agreed nor disagreed with a series of statements. The study instrument is included as Supplementary File 1.

As PREFER aimed to describe participants’ self-reported experiences and preferences, not compare outcomes or test a hypothesis, the sample size for each study site was chosen to optimize the use of study resources. For each study site, we aimed to enroll 100 participants per site. However, we found that there were fewer eligible clients than originally anticipated. We enrolled just over 60% of our total target sample.

### Quantitative Analysis

We first describe participants’ characteristics at enrolment, experience with HIV care, and self-reported preferences on how HIV treatment should be delivered during the first six months of ART treatment using frequencies and simple proportions. Medians with interquartile ranges are reported for continuous variables. For clients’ perceptions of clinical care, which used the 5-point Likert scale described above, we report the proportion of participants who agreed, disagreed, or neither agreed nor disagreed with each of the statements stratified by time on ART and country. To discern differences between newly initiating clients and those who had been on treatment for up to 6 months at study enrolment, we dichotomized participants as either 1) newly initiating or re-initiating ART or 2) as $\le$ 6 months on ART, based on self-report. Where relevant, we further stratified the first group into ART naïve (newly initiative) and experienced (re-initiating) participants, also based on self-report. All results are presented stratified by country.

### Ethics Statement

Country-specific protocols for the PREFER study were approved by Boston University Institutional Review Board under protocol H-42726 (PREFER-South Africa) and H-42903 (PREFER-Zambia). Both protocols were also approved by the University of Witwatersrand Human Research Ethics Committee under protocols M220440 (PREFER-South Africa) and M210342 (PREFER-Zambia). In addition, the protocol for South Africa was approved by the Provincial Health and Research Committees through each study district’s National Health Research Database. The Zambia protocol was also approved by national ethics governing boards, ERES-Converge IRB (2022-June-007) and the Zambia National Health Research Authority (NHRA000007/10/07/2022).

## Results

### Participants’ Characteristics

**Fig. 1:**
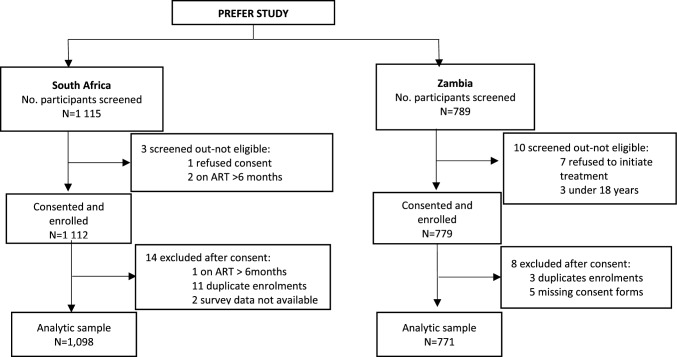
PREFER study enrolment flow chart

Between 7 September 2022 and 30 June 2023, we screened 1115 prospective participants in South Africa and 789 in Zambia (Fig. 1). Participants under 18 years old, those who refused consent, individuals on ART for over six months, and those who declined to initiate treatment were screened out from the study. The final sample considered for analysis after the exclusions listed in Fig. 1 was 1098 for South Africa (72% female) and 771 for Zambia (67% female) in the study. Over a third of participants in South Africa (38%) and Zambia (34%) were initiating or reinitiating ART on the day of study enrolment; the remainder had been on treatment for 0–6 months (Table 1).

**Table 1:** Characteristics of PREFER study participants by country

Characteristics (n, %)	South Africa	Zambia
N	1098	771
Age, median (IQR)	33 (27, 41)	32 (27,40)
Female	786 (72)	514 (67)
Marital status		
Live with a primary partner/spouse	347 (32)	362 (47)
Primary partner/spouse but do not live together	505 (46)	123 (16)
No primary partner/spouse	246 (22)	286 (37)
Literacy level		
Read well	834 (76)	335 (43)
Read somewhat	218 (20)	235 (30)
Cannot read	46 (4)	201 (26)
Highest level of education		
Primary or less	409 (37)	386 (50)
Secondary	525 (48)	329 (43)
Post-secondary	164 (15)	56 (7)
Residence location (based on facility setting)		
Rural	506 (46)	623 (81)
Urban	592 (54)	148 (19)
Considers house of current residence to be main house		
Yes	735 (67)	738 (96)
Main house is somewhere else in South Africa/Zambia	240 (22)	33 (4)
Main house is in another country	123 (11)	-
Employment status		
Formal employment	240 (22)	73 (9)
Informal employment	216 (20)	413 (54)
Unemployed	562 (51)	267 (35)
Student/Trainee	80 (7)	18 (2)
Access to electricity in house (yes)	1060 (97)	524 (68)
Access to piped water		
No	58 (5)	205 (27)
Yes, at house	736 (67)	301 (39)
Yes, at community tap/pipe	304 (28)	265 (34)
Frequency of HH members going without food		
Never	772 (70)	260 (34)
Seldom	72 (7)	91 (12)
Sometimes	220 (20)	352 (46)
Often	34 (3)	68 (9)
Would have difficulty obtaining 100 Rands/100 Kwacha for medical treatment (yes)	615 (56)	629 (82)
Household members who have HIV (self-report)		
No other HH members with HIV	661 (60)	453 (59)
One other HH member with HIV	322 (29)	252 (33)
Two or more other HH members with HIV	115 (10)	66 (8)
If other HH members have HIV, number known to be on ART		
None	64 (15)	49 (15)
One	285 (65)	227 (71)
Two or more	88 (20)	42 (13)
Time on ART at study enrollment		
0 days (initiating or re-initiating on day of enrollment)	419 (38)	261 (34)
1–180 days	679 (62)	510 (66)

*HH* Household

Table 1 reports the demographic characteristics of enrolled participants, stratified by country. The median age was 33 years in South Africa and 32 years in Zambia. Three quarters (76%) of South African participants were literate and 63% had completed primary school. In Zambia 43% of participants reported themselves as literate and half had completed primary school. More than half of South African participants lived in urban areas (54%), while the majority of Zambian participants were rural residents (81%). About half of South African participants said they were unemployed (51%), as did about one third of Zambian participants (35%). Nearly all South African participants had electricity (97%), two thirds (67%) had access to piped water at the house, and 70% reported household members have never gone without food. In Zambia, 68% had electricity, 39% of access to piped water at the house, and 55% reported that they sometimes or often go without food. In both countries, the majority of participants said that it would be difficult or very difficult for them to come up with the equivalent of $5–6 for a medical emergency (56% South Africa, 82% Zambia). In both countries, 60% of participants said that they did not know of any other members of their households who were HIV-positive. Of those who did report other household members with HIV, 85% in South Africa and 84% in Zambia indicated that at least one of these was on ART. In both countries, roughly two thirds of participants (South Africa 62%, Zambia 66%) had been on treatment for between 1 day and 6 months on the day of study enrollment, while the rest reported they were initiating ART that day.

Participants’ characteristics are stratified by time on ART (initiated on the day of survey or initiated 0–6 months before the survey) and sex in Supplementary Table 1. There were few differences in any characteristics by time on ART. A third (South Africa 35%, Zambia 31%) of participants initiating or re-initiating ART on the day of the survey reported knowing of any other household members with HIV.

### Experience with HIV Care

About half of participants (45% South Africa, 54% Zambia) reported that they had tested for HIV because of ill health (Table 2). One third (34%) of South African participants and 18% of Zambian participants said they had tested HIV-positive before the day on which they started ART, but only 12% in South Africa and 2% in Zambia self-reported previous use of ART (Table 2). As we will discuss later, we speculate that the true proportion of re-initiators was much higher, as self-report is not a reliable indicator [29], and the study did not have access to any external data sources to verify naive or non-naive status among participants. Among women enrolled in the study, 9% in South Africa and 5% in Zambia reported that their primary reason for testing was pregnancy or antenatal care.

**Table 2:** PREFER study participants’ prior experience with HIV testing and care

Experience, n (%)	South Africa	Zambia
N	1098	771
Previously tested positive for HIV before starting ART this time	378 (34)	135 (18)
Previously exposed to ART (prior to current treatment experience)	134 (12)	12 (2)
Reason for testing at time of first positive test		
Recommended by healthcare provider	239 (22)	141 (18)
Known exposure or risk	121 (11)	103 (13)
Ill health	490 (45)	413 (54)
Pregnancy/antenatal	67 (6)	27 (4)
Self-initiated/voluntary testing	155 (14)	69 (9)
Other	26 (3)	18 (2)
Time between testing positive and treatment initiation		
Same day	831 (76)	597 (77)
Within a week	186 (17)	111 (14)
Month plus	72 (7)	60 (8)
Can’t remember	9 (1)	3 (0)
How many months of HIV medications did you receive today?		
None	31 (3)	34 (4)
< One month	2 (0)	28 (4)
One month	896 (82)	274 (36)
Two months	114 (10)	59 (8)
Three months	49 (4)	334 (43)
Four months	–	4 (0)
Six months	–	12 (2)
Other	6 (1)	26 (3)

Among participants who were initiating or reinitiating treatment on the day of study enrolment, 93% and 65% in South Africa and Zambia, respectively received a one-month supply of medications at that visit (Supplementary Tables 2 and 3). Dispensing intervals increased after the initiation visit in Zambia, however: 57% of those who had started any time prior to the day of study enrolment received a three-month supply (Supplementary Table 3). This change did not occur in South Africa, where 75% of those who had started prior to study enrolment reported still receiving just a one-month supply (Supplementary Table 2).

### Preferences for HIV Treatment Delivery During the First Six Months of ART Treatment

Table 3 presents study participants’ stated preferences for how they would like to receive care if choices were offered. Only 6% of participants in South Africa and 13% in Zambia said that they had been offered any choices of service delivery locations or dispensing durations. There were no significant or programmatic differences in care preferences by sex or residence (urban v rural) (Supplementary Tables 2 and 3).

**Table 3:** PREFER study participants’ preferences for treatment in the first six months, South Africa and Zambia

Preference, n (%)	South Africa	Zambia
N	1098	771
*Preferred visit and dispensing procedures*		
Offered choice about service delivery since starting ART	68 (6)	100 (13)
Preference for clinic visit frequency		
Every month	140 (13)	61 (8)
Every 2 months	277 (25)	72 (9)
Every 3 months	476 (43)	233 (30)
Every 6 months	190 (17)	372 (48)
Other	15 (1)	33 (4)
Preferred part of the month to visit clinic		
Early in the month (first week)	332 (30)	313 (41)
Late in the month (last week)	171 (16)	187 (24)
Middle of the month	169 (15)	96 (12)
No preference	426 (39)	175 (23)
Preferred time(s) of day to come to clinic visits		
Before work (before 8 am)	392 (36)	202 (26)
Mornings (8 am to 12 pm)	593 (54)	429 (56)
Lunch time (12 am to 2 pm)	101 (9)	41 (5)
Afternoons (2 to 4 pm)	104 (9)	75 (10)
After work (4–7 pm)	42 (4)	58 (8)
Weekends	50 (5)	15 (2)
Other	16 (1)	19 (2)
Preference for bringing companion (friend, family, support person) to clinic	114 (10)	151 (20)
Preference for medications supply to be dispensed		
1 month at a time	125 (11)	39 (5)
2 months at a time	276 (25)	59 (8)
3 months at a time	487 (44)	237 (31)
4 months at a time	16 (1)	17 (2)
6 months at a time	194 (18)	419 (54)
Preference for medication packaging		
One bottle for each month	478 (44)	270 (35)
One large bottle with several months in it	170 (15)	277 (36)
Unmarked (blank) container	78 (7)	53 (7)
Container with instruction label	78 (7)	84 (11)
Blister pack	147 (13)	41 (5)
Any kind of packaging is fine	143 (13)	41 (5)
Something else(specify)	4 (0)	5 (1)
*Preferred provider cadre and service location*		
Preferred care provider		
Doctor or clinical officer	160 (15)	382 (50)
Nurse	872 (79)	200 (26)
Counsellor	52 (5)	158 (20)
CHW, peer/expert patient	7 (1)	14 (2)
Someone else (specify)	7 (1)	17 (2)
Preference for community-based treatment (e.g. at a school, church, or pharmacy)	710 (65)	232 (30)
Preference for home-based treatment	580 (53)	439 (57)
Counselling and education preferences		
Preference for quantity of one-on-one counselling to help manage your treatment, compared to what you received		
More	535 (49)	372 (48)
Same	540 (49)	369 (48)
Less	23 (2)	30 (4)
Preference for quantity of information and education about HIV and ART, compared to what you received		
More	530 (48)	372 (48)
The same	538 (49)	369 (48)
Less	30 (3)	30 (4)
Preferred format to receive information about HIV and ART**		
Written material (brochure or information sheet)	401 (37)	171 (22)
Class/group session in community	89 (8)	51 (7)
Class/group session with provider at clinic	191 (17)	131 (17)
One-on-one session with provider at clinic	543 (49)	350 (45)
Social media (e.g. Facebook, Twitter)	241 (22)	55 (7)
Group within my community	43 (4)	19 (2)
Radio or TV	262 (24)	183 (24)
Videos to watch online at home	112 (10)	13 (2)
Text messages on my phone	589 (54)	236 (31)
Links to websites that I can browse in my own time	194 (18)	6 (1)
Other	3 (0)	4 (0)
Preferred language for receiving information about HIV and ART		
English	422 (38)	128 (17)
isiZulu	301 (27)	
Xitsonga	74 (7)	
Siswati	165 (15)	
Nyanja		346 (45)
Bemba		217 (28)
Tonga		55 (7)

*I/RI = Initiating ART on the day of PREFER study enrollment; $\le$ 6 mos = On ART for 6 months or less at time of study enrollment

**Clients could select as many as apply, so %s > 100%

*CHW* community health worker

### Preferences for Clinic Visits and Medication Dispensing

Clients in South Africa frequently expressed preferences for 2-month (25%) or 3-month (43%) visit frequency; fewer preferred 6-month visits (17%). In contrast, Zambian clients expressed preferences for less frequent visit scheduling, with 48% stating that they would prefer 6-monthly visits. Similarly, clients in South Africa preferred 2 months (25%) or 3 months (44%) of medication to be dispensed at a time, while Zambian participants more often preferred 6-month dispensing (54%). Few Zambian participants (13%) expressed preferences for shorter 1- or 2-month dispensing intervals. Clients in both countries preferred morning visits (before work or before 12 pm) and most often wanted to visit the clinic alone, instead of with a companion or support person. Participants generally preferred large bottles to store their medication, with either one large bottle for each month, or one bottle to store several months of medication supply.

### Preferred Provider Cadre and Service Delivery Location

In South Africa, 79% of participants preferred to receive care from a nurse; in Zambia half (50%) preferred to receive care from a doctor or clinical officer. Very few clients preferred care from a community health worker, peer expert, or expert patient; more Zambian participants (20%) opted for care from a counselor than did South African participants (5%). Two thirds of clients in South Africa (65%) were receptive to service delivery in a community setting, such as a school, church, or pharmacy, and 53% preferred home delivery. Community-based delivery was less preferred in Zambia, where 30% indicated this preference.

### Counselling and Health Education

Just under half of participants in both countries expressed a preference for more one-on-one counseling than they said they had received (South Africa: 49%; Zambia: 48%). Other participants opted for the same amount of counseling they received; less than 5% of the participants wanted less counseling. Trends were similar in both countries when asked about the quantity of information and education they received about HIV and ART: most clients wanted the same or more information. Stratified analysis (Supplementary Tables 2 and 3) showed some variation but few important differences by age group or sex.

Participants varied in their preferences of formats in which to receive information about HIV and ART. In both countries, nearly half of the participants (South Africa 49%, Zambia 45%) indicated a preference for one-on-one sessions with providers in the clinic and 54% and 31% preferred informational text messages, respectively. In South Africa, there was also a preference for written brochures or flyers (37%), while others in Zambia preferred radio/television programming (24%).

### Differences Between New Initiators and Re-initiators

As shown in Table 2, very few Zambian participants (n = 12, 2% of sample) voluntarily disclosed previous exposure to ART, but a more substantial proportion in South Africa (n = 134, 12%) reported prior treatment experience in the survey responses. In South Africa, another data field in the survey (reasons for the clinic visit allowed us to identify an additional 34 South African clients whose reason for clinic visit indicated current ART treatment (e.g. medication refill), generating a total of 168 clients with evidence of prior treatment experience. In Zambia an additional of 30 clients were identified from other data fields, giving a total of 42 clients with previous treatment experience. Table 4 describes results from the sample restricted to study participants initiating or re-initiating treatment on the day of study enrollment who have evidence of prior treatment experience (re-initiators) with those who self-reported as naïve initiators and for whom no other evidence exists. In both countries, absolute numbers of known re-initiators are small, and this should be considered in interpreting the results in Table 4.

**Table 4:** PREFER study participants’ preferences for treatment in the first six months by ART naïve (newly initiating) and experienced (re-initiating) status, South Africa and Zambia

Preference, n (%)	South Africa			Zambia		
	Total	Naïve	Reinitiating	Total	Naïve	Reinitiating
N (initiators and re-initiators only)	479	311	168	268	226	42
*Preferred visit and dispensing procedures*						
Offered choice about service delivery since starting ART	33 (7)	14 (5)	19 (11)	32 (12)	21 (9)	11 (26)
Preference for clinic visit frequency						
Every month	80 (17)	55 (18)	25 (15)	34 (13)	31 (14)	3 (7)
Every 2 months	120 (25)	87 (28)	33 (20)	32 (12)	28 (12)	4 (10)
Every 3 months	202 (42)	127 (41)	75 (45)	81 (30)	64 (28)	17 (40)
Every 6 months	72 (15)	39 (13)	33 (20)	106 (40)	92 (41)	14 (33)
Other	5 (1)	3 (1)	2 (1)	15 (6)	11 (5)	4 (10)
Preferred part of the month to visit clinic						
Early in the month (first week)	137 (29)	83 (27)	54 (32)	109 (41)	88 (39)	21 (50)
Late in the month (last week)	79 (16)	50 (16)	29 (17)	63 (24)	56 (25)	7 (17)
Middle of the month	74 (15)	48 (15)	26 (15)	36 (13)	29 (13)	7 (17)
No preference	189 (39)	130 (42)	59 (35)	60 (22)	53 (23)	7 (17)
Preferred time(s) of day to come to clinic visits						
Before work (before 8 am)	184 (38)	112 (36)	72 (43)	70 (26)	62 (27)	8 (19)
Mornings (8 am to 12 pm)	249 (52)	170 (55)	79 (47)	138 (51)	113 (50)	25 (60)
Lunch time (12 am to 2 pm)	39 (8)	24 (8)	15 (9)	13 (5)	10 (4)	3 (7)
Afternoons (2 to 4 pm)	55 (11)	38 (12)	17 (10)	30 (11)	24 (11)	6 (14)
After work (4–7 pm)	22 (5)	18 (6)	4 (2)	26 (10)	25 (11)	1 (2)
Weekends	24 (5)	20 (6)	4 (2)	7 (3)	7 (3)	0 (0)
Other	9 (2)	4 (1)	5 (3)	6 (2)	6 (3)	0 (0)
Preference for bringing companion (friend, family, support person) to clinic	56 (12)	36 (12)	20 (12)	63 (24)	57 (25)	6 (14)
Preference for medication quantity dispensed						
1 month at a time	68 (14)	47 (15)	21 (12)	26 (10)	24 (11)	2 (5)
2 months at a time	121 (25)	87 (28)	34 (20)	26 (10)	22 (10)	4 (10)
3 months at a time	211 (44)	134 (43)	77 (46)	84 (31)	67 (30)	17 (40)
4 months at a time	8 (2)	5 (2)	3 (2)	6 (2)	5 (2)	1 (2)
6 months at a time	71 (15)	38 (12)	33 (20)	126 (47)	108 (48)	18 (43)
Preference for medication packaging						
One bottle for each month	226 (47)	143 (46)	83 (49)	87 (32)	72 (32)	15 (36)
One larger bottle with several months in it	71 (15)	46 (15)	25 (15)	85 (32)	70 (31)	15 (36)
An unmarked (blank) container	32 (7)	20 (6)	12 (7)	22 (8)	19 (8)	3 (7)
A container with instructions on it	36 (8)	27 (9)	9 (5)	38 (14)	32 (14)	6 (14)
A blister pack	57 (12)	35 (11)	22 (13)	23 (9)	20 (9)	3 (7)
Any kind of packaging is fine	54 (11)	38 (12)	16 (10)	12 (4)	12 (5)	0 (0)
Something else	3 (1)	2 (1)	1 (0)	1 (0)	1 (0)	0 (0)
Preferred provider cadre						
Doctor or clinical officer	83 (17)	47 (15)	36 (21)	125 (47)	105 (46)	20 (48)
Nurse	369 (77)	242 (78)	127 (76)	64 (24)	51 (23)	13 (31)
Counsellor	23 (5)	18 (6)	5 (3)	72 (27)	64 (28)	8 (19)
Community health worker/peer expert	3 (1)	3 (1)	0 (0)	3 (1)	2 (1)	1 (2)
Someone else	1 (0)	1 (0)	0 (0)	4 (1)	4 (2)	0 (0)
Preferred location of care						
Community-based (e.g. at a school or church or pharmacy)	307 (64)	196 (63)	111 (66)	78 (29)	64 (28)	14 (33)
Home medication delivery	268 (56)	168 (54)	100 (60)	158 (59)	132 (58)	26 (62)
*Counselling and health education preferences*						
Preference for more/ less one-on-one counselling to help manage your treatment						
More	250 (52)	161 (52)	89 (53)	135 (50)	109 (48)	26 (62)
The same	217 (45)	141 (45)	76 (45)	125 (47)	109 (48)	16 (38)
Less	12 (3)	9 (3)	3 (2)	8 (3)	8 (4)	0 (0)
Preference for more/less information and education about HIV and ART						
More	236 (49)	149 (48)	87 (52)	136 (51)	111 (49)	25 (60)
The same	229 (48)	151 (49)	78 (46)	123 (46)	106 (47)	17 (40)
Less	14 (3)	11 (4)	3 (2)	9 (3)	9 (4)	0 (0)
Preferred format to receive information about HIV and ART**						
Written material (brochure or information sheet)	163 (34)	112 (36)	51 (30)	69 (26)	55 (24)	14 (33)
Class/group session in community	49 (10)	32 (10)	17 (10)	21 (8)	19 (8)	2 (5)
Class/group session with provider at clinic	106 (22)	61 (20)	45 (27)	39 (15)	32 (14)	7 (17)
One-on-one session with provider at clinic	244 (51)	160 (51)	84 (50)	117 (44)	105 (46)	12 (29)
Social media (e.g. Facebook, Twitter)	109 (23)	58 (19)	51 (30)	21 (8)	18 (8)	3 (7)
Group within my community	23 (5)	15 (5)	8 (5)	9 (3)	8 (4)	1 (2)
Radio or TV	127 (27)	76 (24)	51 (30)	64 (24)	54 (24)	10 (24)
Videos to watch online at home	45 (9)	27 (9)	18 (11)	2 (1)	1 (0)	1 (2)
Text messages on my phone	249 (52)	164 (53)	85 (51)	86 (32)	75 (33)	11 (26)
Links to websites that I can browse in my own time	74 (15)	51 (16)	23 (14)	2 (1)	2 (1)	0 (0)
Other	3 (1)	2 (1)	1 (0)	3 (1)	3 (1)	0 (0)
Preferred language for receiving information about HIV and ART, n (%)						
English	178 (37)	124 (40)	54 (32)	42 (16)	37 (16)	5 (12)
isiZulu	102 (21)	65 (21)	37 (22)	-	-	-
Xitsonga	43 (9)	30 (10)	13 (8)	-	-	-
Siswati	91 (19)	60 (19)	31 (18)	-	-	-
Nyanja	-	-	-	110 (41)	90 (40)	20 (48)
Bemba	-	-	-	87 (32)	74 (33)	13 (31)
Tonga	-	-	-	20 (7)	16 (7)	4 (10)

Sample for Table 4 is limited to participants initiating or re-initiating ART at the time of study enrollment; those already on ART (initiated up to 6 months previously) are not included here

Differences between naïve and re-initiators were small or non-existent for most preferences. Re-initiators were slightly more likely to report having been given a choice of service delivery characteristics. Preferences for visit and dispensing intervals and timing, medication quantities and packaging, and provider and service location did not differ in any consistent or meaningful way. Re-initiating participants in Zambia were slightly more likely than South African participants to favor more counseling, information, and education than they received, but differences were modest.

## Clients’ Views on Service Delivery

**Fig. 2:**
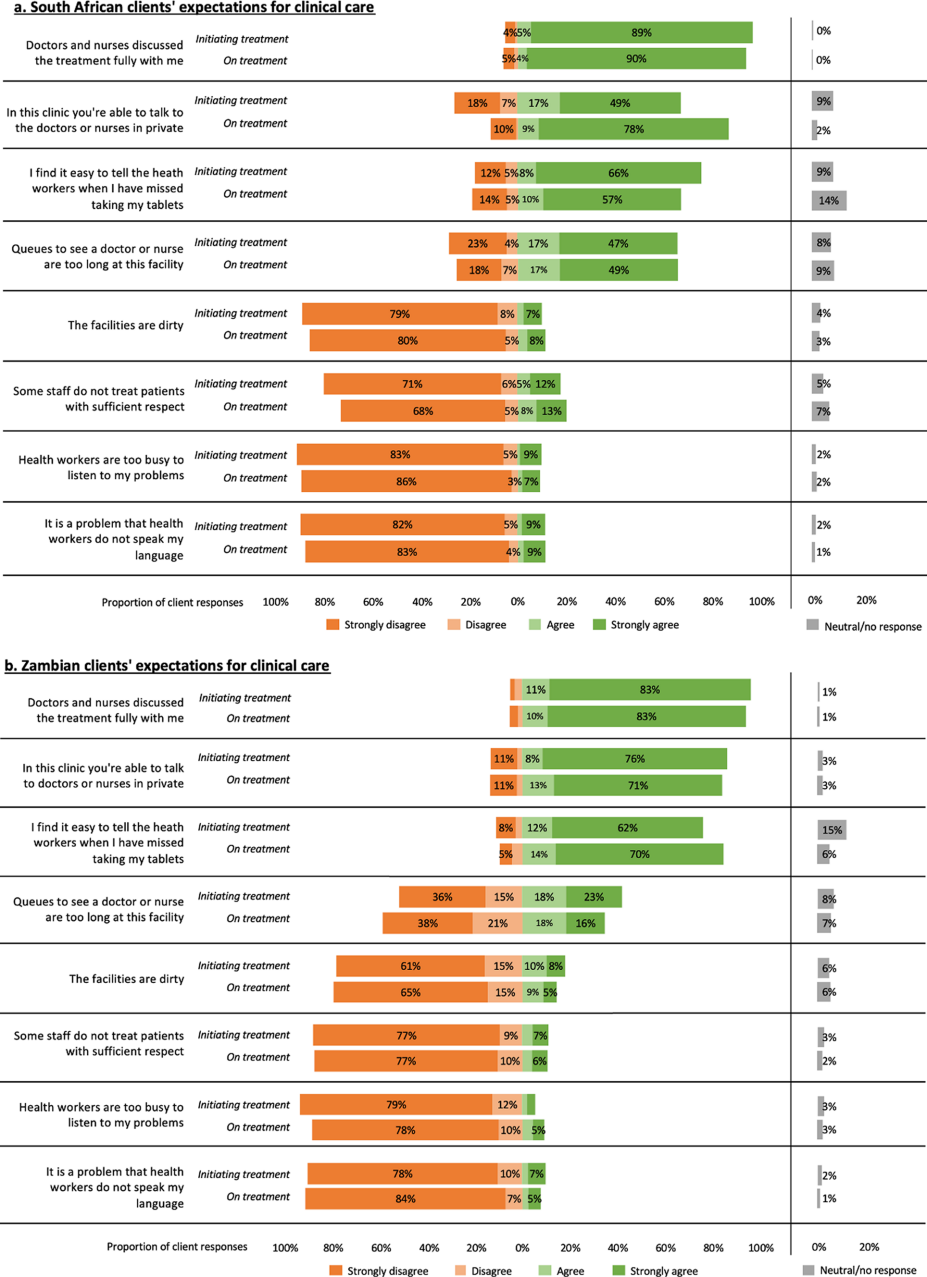
Participants’ views of services received in (a) South Africa and (b) Zambia

Figure 2 presents survey participants’ reported views on key aspects of HIV treatment delivery at the study sites stratified by country and time on ART (initiating or re-initiating on day of survey enrollment or initiating up to 6 months before survey enrollment). Results were largely but not entirely positive, with regard to views on clinic staff and facilities. Most of the participants in both countries, regardless of their time on ART, strongly agreed that the doctors and nurses had discussed the treatment fully, that they were able to talk with them privately, and that it was easy to tell healthcare providers that they had missed tablets, and most strongly disagreed that providers were too busy to listen to their problems. Across both countries and treatment groups, between 10 and 19%, noted difficulty in mentioning missed tablets. More than two thirds of the participants for both treatment groups in South Africa and roughly 40% of those in Zambia strongly or mildly agreed that the queues to see a provider were too long. In Zambia, more than half of the participants disagreed with this statement, generally not perceiving the queues to be too long.

## Discussion

In this survey of 1,869 ART clients in South Africa and Zambia during the first six months after initiation or re-initiation of treatment, when they were not eligible for existing DSD models, we found that few choices were offered with regard to the manner of service delivery. This lack of options is consistent with current guidelines, but it may not reflect client preferences and thus may indicate an opportunity for improving early outcomes.

Notably, clients in both countries preferred to receive multi-month medication dispensing and have less frequent visit requirements during the first 6 months on ART. In Zambia, the vast majority of all participants—more than 85%–preferred three or more months of medication dispensed at a time, but 36% actually received only one month at a time. Nearly two thirds of clients in South Africa would prefer 3 or 6 monthly clinic visits with 3–6 months of medications dispensed at a time but, again, guidelines did not allow this for the early treatment period. An unintended consequence of requiring frequent visits and medication refills in the early treatment period is that a client’s early experiences establish their expectations for what treatment will be like going forward. If new clients discover that service delivery is burdensome and inflexible during the early treatment period, they may be deterred from remaining in care until they are eligible for lower-burden DSD models.

Previous reports among clients already eligible for DSD (i.e. on ART for more than 6 months) in southern African countries have indicated some similar findings to ours: clients preferred less frequent facility-based visits and shorter wait times [15–17, 20]. Contrary to findings in some other studies among established ART patients in sub-Saharan Africa [15, 16], however, most participants in our survey in both countries reported positive experiences at facilities, particularly with regard to interactions with healthcare providers. Clients generally had positive perspectives of the clinics at which they sought care, expressing trust in the facility, attesting that their providers discussed treatment options with them, and feeling that they had adequate privacy, were comfortable discussing adherence challenges, felt respected, and had their concerns addressed. The reason for this discrepancy between our results and previously published findings is unclear, but it may reflect different provider or client behavior or expectations at initiation and during the early treatment period, compared to later in their treatment journey. It is also possible that the earlier published research revealing the poor regard in which clients held healthcare providers and the importance of better client-provider relationships has in fact improved interactions, and that these improvements are reflected in our more recent data set.

At the same time, many participants commented on long waiting times at facilities and problems retrieving client records, particularly in South Africa. These concerns may reduce motivation to attend future visits [20], especially if visits are required monthly, as is the case in many countries during the early treatment period [12]. In fact, long waiting times were the only facility characteristic that elicited negative reactions from a large minority of study participants.

We were surprised to find that roughly half of participants in both countries expressed a preference for more counseling and information than they received, and virtually none wanted less, despite published doubts about the quality of counseling offered [30]. This was consistent regardless of previous treatment experience and time on ART, suggesting that emotional support and information dissemination are important needs throughout the early treatment period, for all types of clients. Many clients also said that they would like more one-on-one counseling, with nearly half indicating that their preferred method to receive information was through one-on-one sessions with their provider in the health facility. This may present a challenge to improving early treatment outcomes, as anecdotal evidence from treatment providers in both South Africa and Zambia points to reductions in the human resources available to provide counselling and treatment literacy services.

One solution to the discrepancy between the amount and types of counseling that clients reported wanting and what the healthcare system can reasonably offer may be greater use of telehealth options. A substantial proportion of participants in South Africa indicated willingness to receive information through online or remote interaction modalities like social media and links to websites, which could reduce the burden on facility-based services and empower clients to more wholly participate in their care. In Zambia, where internet connectivity is less widespread than South Africa, text messaging support and radio or television broadcasts may be a better approach. While it was once thought that group-based models, such as adherence clubs, would provide the emotional support desired, we speculate that COVID-19 fears and multi-month dispensing, combined with ongoing stigma and fear of disclosure of HIV status, have reduced demand for these models, and most study participants in both countries indicated a preference for individualized counselling over group-based models. Going forward, there may be a renewed role for peer-based support services after ART initiation to replace support that was previously offered by a more robust counseling cadre [31].

In our study, while client preferences were often diverse, stratifying clients by time on ART, gender, and naïve vs. non-naïve status did not result in significant difference in preferences or perceptions of care. Much of the published literature suggests that men’s and women’s care needs and uptake behaviors differ [17, 32], but we found that preferences between men and women were not meaningfully different. We also found no important or programmatically meaningful differences in preferences between self-reported naïve clients and those re-initiating after a period of disengagement. Individuals within all the key strata—sex, age, prior treatment experience–expressed varied views, with few unanimous or subgroup-specific preferences. While we were able to identify aspects of care that were more or less favored by some subgroups, there were seldom overwhelming majority preferences among our sample.

Based on our findings, we speculate that there other characteristics of subgroups–prior experience with healthcare overall, biases, preconceived notions about HIV, social support, readiness to begin treatment [33], other emotional and personal factors–may affect care preferences more than concrete demographic factors. One implication of this result is that the development of accurate risk stratification approaches to identify individuals at risk of negative outcomes who might require different services, which we believe is a promising direction for further research [34, 35], may be even more challenging than expected [24, 25]. If self-reported HIV/ART history and basic demographic characteristics do not provide sufficient information to determine optimal service delivery models, the role of self-identification of risk and individual choice of service delivery characteristics may become more important, allowing clients to self-select what is best for them [19, 22]^23^.

Finally, we note that for several of the preferences reported, differences observed between the two countries appear to reflect existing conditions with which clients are familiar. In South Africa, participants preferred two- or three-month intervals between visits and two- or three-month medication dispensing, with care provided by a nurse; this was the standard of care at the time of the survey. In Zambia, where national guidelines allowed 6-month dispensing once a client is eligible for DSD and most clinical consultations are provided by clinical officers, participants said they preferred 6-month dispensing and care from clinical officers. It is thus unclear what clients’ true preferences would be in the absence of clinical norms and experience [15, 16].

Our study had both a number of strengths and several limitations. Its major strength is its focus on the early treatment period; as noted earlier, to our knowledge there are no prior studies that explore preferences among ART clients in their first six months on treatment, before they are eligible for DSD models. The inclusion of two high HIV-prevalence countries and multiple geographic locations is also a strength. Under limitations, we note that the sample size was modest and included only a handful of facilities in each country, potentially limiting generalizability. As with any survey, results are based on self-report, which may or may not reflect actual experience or views. This is particularly important when comparing ART naïve to re-initiating clients, as there is evidence that non-disclosure of prior treatment experience is common [29]. Our ability to distinguish naive and non-naive initiators was poor, and comparisons of these populations must be interpreted with caution. In addition, this study was conducted prior to the adoption of new HIV care guidelines in South Africa. The recently adopted guidelines for South Africa allow eligibility for DSD models as early as four months after treatment initiation for those with a suppressed viral load, rather than the previous six months [36]. Finally, we assume that our results reflect some degree of social desirability bias, leading to more positive responses to some questions than might be truly representative of participants’ views.

## Conclusion

In many countries, the early treatment period remains a challenge for delivering HIV treatment in a way that supports retention in care and other positive outcomes. While we did not identify a “smoking gun” to explain the high rates of interruption and disengagement observed during this period, we did find a wide range of preferences that can be used to improve specific aspects of service delivery for specific subgroups of the population. We also found that the preferences of clients in the early treatment period align with existing DSD models designed for established clients, such as multi-month dispensing in Zambia and community-based delivery (external pickup points) in South Africa. Existing models may thus offer a strong base on which to build more effective models for the early treatment period. New treatment guidelines in South Africa, which allow enrollment in lower-intensity models of care as early as four months after initiation, are a step in this direction [11]. Allowing longer dispensing intervals and fewer clinic visits immediately after initiation may be feasible, at least for clients without clinical conditions requiring active care, and should be considered as an option that could be offered by providers. In Zambia, where six-month dispensing and infrequent clinic visits are already the norm for those with more than six months' experience on ART, offering this option to initiating clients, perhaps with follow-up by telephone or community health worker visits, may also help align guidelines with users' preferences. Analysis of Zambian electronic medical records in previously published work suggests that informal early enrollment in a range of DSD models does not harm health outcomes [14]. Future research will examine the feasibility of adjusting guidelines to allow earlier enrollment in existing DSD models in both countries.

## Supplementary Information

Below is the link to the electronic supplementary material.

Supplementary file1 (DOCX 50 kb)

Supplementary file1 (PDF 51 kb)

## Abbreviations

ART: Antiretroviral therapy
DSD: Differentiated service delivery
HH: Household
ITT: Interruptions to treatment
