# Supplementary material for: Experiences and Preferences in Zambia and South Africa for Delivery of HIV Treatment During a Client’s First Six Months: Results of the PREFER Study’s Cross-Sectional Baseline Survey
Source: AIDS Behav. 2025 Feb 1;29(6):1713–28. doi: 10.1007/s10461-025-04640-y (PMC12075270; doi:10.1007/s10461-025-04640-y)
Supplement: Supplementary file 1 — Supplementary file1 (DOCX 50 kb) [file 10461_2025_4640_MOESM1_ESM.docx]

**Supplementary Tables**

Supplementary Table 1. Participant characteristics by country, sex, and time on treatment at enrolment

| **Characteristic (n, %)** | **South Africa** | | | | | **Zambia** | | | | |
| --- | --- | --- | --- | --- | --- | --- | --- | --- | --- | --- |
|  | **Total** | **Sex** | | **Time on ART at enrolment** | | **Total** | **Sex** | | **Time on ART at enrolment** | |
|  |  | **Male** | **Female** | **I/RI*** | **≤6 mos** |  | **Male** | **Female** | **I/RI*** | **≤6 mos** |
| N | 1,098 | 312 (28) | 786 (72) | 419 (38) | 679 (62) | 771 | 257 (33) | 514 (67) | 261 (34) | 510 (66) |
| Age, median (IQR) | 33 (27, 41) | 38 (33, 44) | 31 (25, 39) | 34 (27,40) | 33 (27,41) | 32 (27,40) | 36 (30, 43) | 31 (25, 39) | 32 (27,40) | 33 (27,40) |
| Female | 786 (72) |  |  | 289 (69) | 497 (73) | 514 (67) |  |  | 151 (58) | 363 (71) |
| Marital status |  |  |  |  |  |  |  |  |  |  |
| *Live with a primary partner/spouse* | 347 (32) | 131 (42) | 216 (27) | 137 (33) | 210 (31) | 362 (47) | 141 (55) | 221 (43) | 126 (48) | 236 (46) |
| *Primary partner/spouse but do not live together* | 505 (46) | 119 (38) | 386 (49) | 180 (43) | 325 (48) | 123 (16) | 40 (16) | 83 (16) | 40 (15) | 83 (16) |
| *No primary partner/spouse* | 246 (22) | 62 (20) | 184 (23) | 102 (24) | 144 (21) | 286 (37) | 76 (30) | 210 (41) | 95 (36) | 191 (37) |
| Literacy level |  |  |  |  |  |  |  |  |  |  |
| *Read well* | 834 (76) | 223 (71) | 611 (78) | 314 (75) | 520 (77) | 335 (43) | 136 (53) | 199 (39) | 125 (48) | 210 (41) |
| *Read somewhat* | 218 (20) | 72 (23) | 146 (19) | 89 (21) | 129 (19) | 235 (30) | 71 (28) | 164 (32) | 70 (27) | 165 (32) |
| *Cannot read* | 46 (4) | 17 (5) | 29 (4) | 16 (4) | 30 (4) | 201 (26) | 50 (19) | 151 (29) | 66 (25) | 135 (26) |
| Highest level of education |  |  |  |  |  |  |  |  |  |  |
| *Primary or less* | 409 (37) | 131 (42) | 278 (35) | 147 (35) | 262 (39) | 386 (50) | 113 (44) | 273 (53) | 125 (48) | 261 (51) |
| *Secondary* | 525 (48) | 147 (47) | 378 (48) | 213 (51) | 312 (46) | 329 (43) | 119 (46) | 210 (41) | 115 (44) | 214 (42) |
| *Post-secondary* | 164 (15) | 34 (11) | 130 (17) | 59 (14) | 105 (15) | 56 (7) | 25 (10) | 31 (6) | 21 (8) | 35 (7) |
| Considers house of current residence to be main house |  |  |  |  |  |  |  |  |  |  |
| *Yes* | 735 (67) | 217 (70) | 518 (66) | 279 (67) | 456 (67) | 738 (96) | 247 (96) | 491 (96) | 246 (94) | 492 (96) |
| *No, main house is somewhere else in South Africa/Zambia* | 240 (22) | 58 (19) | 182 (23) | 92 (23) | 148 (22) | 33 (4) | 10 (4) | 23 (4) | 15 (6) | 18 (4) |
| *No, main house is in another country* | 123 (11) | 37 (12) | 86 (11) | 48 (11) | 75 (11) | - | - | - | - | - |
| Employment status |  |  |  |  |  |  |  |  |  |  |
| *Formal employment* | 240 (22) | 108 (35) | 132 (17) | 91 (22) | 149 (22) | 73 (9) | 45 (18) | 28 (5) | 35 (13) | 38 (7) |
| *Informal employment* | 216 (20) | 84 (27) | 132 (17) | 93 (22) | 123 (18) | 413 (54) | 165 (64) | 248 (48) | 135 (52) | 278 (55) |
| *Unemployed* | 562 (51) | 112 (36) | 450 (57) | 201 (48) | 361 (53) | 267 (35) | 43 (17) | 224 (44) | 85 (33) | 182 (36) |
| *Student/Trainee* | 80 (7) | 8 (3) | 72 (9) | 34 (8) | 46 (7) | 18 (2) | 4 (2) | 14 (3) | 6 (2) | 12 (2) |
| Access to electricity in house (yes) | 1060 (97) | 294 (94) | 766 (97) | 405 (97) | 655 (96) | 524 (68) | 157 (61) | 367 (71) | 179 (69) | 345 (68) |
| Access to piped water |  |  |  |  |  |  |  |  |  |  |
| *No* | 58 (5) | 18 (6) | 40 (5) | 20 (5) | 38 (6) | 205 (27) | 68 (26) | 137 (27) | 56 (21) | 149 (29) |
| *Yes, at house* | 736 (67) | 189 (61) | 547 (70) | 283 (68) | 453 (67) | 301 (39) | 94 (37) | 207 (40) | 109 (42) | 192 (38) |
| *Yes, at community tap/pipe* | 304 (28) | 105 (34) | 199 (25) | 116 (28) | 188 (28) | 265 (34) | 95 (37) | 170 (33) | 96 (37) | 169 (33) |
| Frequency of HH members going without food |  |  |  |  |  |  |  |  |  |  |
| *Never* | 772 (70) | 213 (68) | 559 (71) | 295 (70) | 477 (70) | 260 (34) | 102 (40) | 158 (31) | 96 (37) | 164 (32) |
| *Seldom* | 72 (7) | 19 (6) | 53 (7) | 23 (5) | 49 (7) | 91 (12) | 26 (10) | 65 (13) | 27 (10) | 64 (13) |
| *Sometimes* | 220 (20) | 73 (23) | 147 (19) | 91 (22) | 129 (19) | 352 (46) | 111 (43) | 241 (47) | 119 (46) | 233 (46) |
| *Often* | 34 (3) | 7 (2) | 27 (3) | 10 (2) | 24 (4) | 68 (9) | 18 (7) | 50 (10) | 19 (7) | 49 (10) |
| Would have difficulty obtaining 100 Rands/100 Kwacha for medical treatment (yes) | 615 (56) | 170 (54) | 445(56) | 236 (56) | 379 (56) | 629 (82) | 197 (77) | 432 (84) | 214 (82) | 415 (81) |
| Other HH members who have HIV (self-report) |  |  |  |  |  |  |  |  |  |  |
| *No other HH members with HIV* | 661 (60) | 175 (56) | 486 (62) | 271 (65) | 390 (57) | 453 (59) | 152 (59) | 301 (59) | 180 (69) | 273 (54) |
| *One other HH member with HIV* | 322 (29) | 111 (36) | 211 (27) | 118 (28) | 204 (30) | 252 (33) | 86 (33) | 166 (32) | 69 (26) | 183 (36) |
| *Two or more other HH members with HIV* | 115 (10) | 21 (8) | 89 (11) | 30 (7) | 85 (12) | 66 (8) | 60 (23) | 82(16) | 12 (5) | 54 (10) |
| If other HH members have HIV, number known to be on ART |  |  |  |  |  |  |  |  |  |  |
| *None* | 64 (15) | 15 (11) | 49 (16) | 20 (14) | 44 (15) | 49 (15) | 15 (14) | 34 (16) | 12 (15) | 37 (16) |
| *One* | 285 (65) | 101 (74) | 184 (61) | 105 (71) | 180 (62) | 227 (71) | 78 (74) | 149 (70) | 62 (77) | 165 (70) |
| *Two or more* | 88 (20) | 21 (15) | 67 (22) | 23 (15) | 65 (23) | 42 (13) | 12 (11) | 30 (14) | 7 (8) | 35 (14) |

Supplementary Table 2. Participants’ preferences for treatment in the first six months by sex, age, and time on ART at enrolment, South Africa

| **Preference (n, %)** | **Total** | **Sex** | | **Age groups (years)** | | | **Place of Residence** | | **Time on ART**  **at study enrolment** | |
| --- | --- | --- | --- | --- | --- | --- | --- | --- | --- | --- |
|  |  | **Male** | **Female** | **18-24** | **25-49** | **50+** | **Rural** | **Urban** | ***I/RI** | **≤6 mos** |
| N | 1098 | 312 | 786 | 188 | 801 | 109 | 506 | 592 | 419 | 679 |
| ***Preferred visit and dispensing procedures*** |  |  |  |  |  |  |  |  |  |  |
| Offered choice about service delivery since starting ART (yes) | 68 (6) | 21 (7) | 47 (6) | 13 (7) | 48 (6) | 7 (6) | 16 (3) | 52 (9) | 31 (7) | 37 (5) |
| Preference for clinic visit frequency |  |  |  |  |  |  |  |  |  |  |
| *Every month* | 140 (13) | 49 (16) | 91 (12) | 25 (13) | 103 (13) | 12 (11) | 61 (12) | 79 (13) | 73 (17) | 67 (10) |
| *Every 2 months* | 277 (25) | 81 (26) | 196 (25) | 49 (26) | 195 (24) | 33 (30) | 129 (25) | 148 (25) | 107 (26) | 170 (25) |
| *Every 3 months* | 476 (43) | 127 (41) | 349 (44) | 74 (39) | 354 (44) | 48 (44) | 228 (45) | 248 (42) | 174 (42) | 302 (44) |
| *Every 6 months* | 190 (17) | 51 (16) | 139 (18) | 39 (21) | 139 (17) | 12 (11) | 78 (15) | 112 (19) | 62 (15) | 128 (19) |
| *Other* | 15 (1) | 4 (1) | 11 (1) | 1 (1) | 10 (1) | 4 (4) | 10 (2) | 5 (1) | 3 (1) | 12 (2) |
| Preferred part of the month to visit clinic |  |  |  |  |  |  |  |  |  |  |
| *Early in the month (first week)* | 332 (30) | 79 (25) | 253 (32) | 68 (36) | 234 (29) | 30 (28) | 135 (27) | 197 (33) | 120 (29) | 212 (31) |
| *Late in the month (last week)* | 171 (16) | 61 (20) | 110 (14) | 25 (13) | 134 (17) | 12 (11) | 79 (16) | 92 (16) | 65 (16) | 106 (16) |
| *Middle of the month* | 169 (15) | 45 (14) | 124 (16) | 30 (16) | 122 (15) | 17 (16) | 67 (13) | 102 (17) | 67 (16) | 102 (15) |
| *No preference* | 426 (39) | 127 (41) | 299 (38) | 65 (35) | 311 (39) | 50 (46) | 225 (44) | 201 (34) | 167 (40) | 259 (38) |
| Preferred time(s) of day to come to clinic visits |  |  |  |  |  |  |  |  |  |  |
| *Before work (before 8 am)* | 392 (36) | 96 (31) | 296 (38) | 53 (28) | 287 (36) | 52 (48) | 165 (33) | 227 (38) | 161 (38) | 231 (34) |
| *Mornings (8 am to 12 pm)* | 593 (54) | 171 (55) | 422 (54) | 108 (57) | 425 (53) | 60 (55) | 254 (50) | 339 (57) | 224 (53) | 369 (54) |
| *Lunch time (12 am to 2 pm)* | 101 (9) | 27 (9) | 74 (9) | 16 (9) | 73 (9) | 12 (11) | 38 (8) | 63 (11) | 32 (8) | 69 (10) |
| *Afternoons (2 to 4 pm)* | 104 (9) | 29 (9) | 75 (10) | 26 (14) | 68 (8) | 10 (9) | 42 (8) | 62 (10) | 50 (12) | 54 (8) |
| *After work (4-7 pm)* | 42 (4) | 15 (5) | 27 (3) | 10 (5) | 28 (3) | 4 (4) | 16 (3) | 26 (4) | 22 (5) | 20 (3) |
| *Weekends* | 50 (5) | 23 (7) | 27 (3) | 6 (3) | 37 (5) | 7 (6) | 30 (6) | 20 (3) | 23 (5) | 27 (4) |
| *Other* | 16 (1) | 6 (2) | 10 (1) | 3 (2) | 11 (1) | 2 (2) | 14 (3) | 2 (0) | 8 (2) | 8 (1) |
| Preference for bringing companion (friend, family, support person) to clinic (yes) | 114 (10) | 31 (10) | 83 (11) | 23 (12) | 79 (10) | 12 (11) | 50 (10) | 64 (11) | 46 (11) | 68 (10) |
| How many months of HIV medications did you receive today? |  |  |  |  |  |  |  |  |  |  |
| *None* | 31 (3) | 11(4) | 20 (3) | 7 (4) | 21 (3) | 3 (3) | 19 (4) | 12 (2) | 6 (1) | 25 (4) |
| *< One month* | 2 (0) | 1 (0) | 1 (0) | 0 (0) | 2 (0) | 0 (0) | 0 (0) | 2 (0) | 2 (0) | - |
| *One month* | 896 (82) | 241 (77) | 655 (83) | 156 (83) | 648 (81) | 92 (84) | 400 (79) | 496 (84) | 388 (93) | 508 (75) |
| *Two months* | 114 (10) | 42 (13) | 72 (9) | 15 (8) | 93 (12) | 6 (5) | 59 (12) | 55 (9) | 10 (2) | 104 (15) |
| *Three months* | 49 (4) | 14 (4) | 35 (4) | 10 (5) | 34 (4) | 5 (5) | 26 (5) | 23 (4) | 13 (3) | 36 (5) |
| *Other* | 6 (1) | 3 (1) | 3 (0) | 0 (0) | 3 (0) | 3 (3) | 2 (0) | 4 (1) |  | 6 (1) |
| Preference for how many months of medications to be dispensed |  |  |  |  |  |  |  |  |  |  |
| *1 month at a time* | 125 (11) | 44 (14) | 81 (10) | 23 (12) | 89 (11) | 13 (12) | 59 (12) | 66 (11) | 61 (15) | 64 (9) |
| *2 months at a time* | 276 (25) | 83 (27) | 193 (25) | 46 (24) | 199 (25) | 31 (28) | 128 (25) | 148 (25) | 108 (26) | 168 (25) |
| *3 months at a time* | 487 (44) | 128 (41) | 359 (46) | 75 (40) | 362 (45) | 50 (46) | 231 (46) | 256 (43) | 183 (44) | 304 (45) |
| *4 months at a time* | 16 (1) | 5 (2) | 11 (1) | 4 (2) | 11 (1) | 1 (1) | 8 (2) | 8 (1) | 7 (2) | 9 (1) |
| *6 months at a time* | 194 (18) | 52 (17) | 142 (18) | 40 (21) | 140 (17) | 14 (13) | 80 (16) | 114 (19) | 60 (14) | 134 (20) |
| Preference for medication packaging |  |  |  |  |  |  |  |  |  |  |
| *One bottle for each month* | 478 (44) | 144 (46) | 334 (42) | 78 (41) | 346 (43) | 54 (50) | 230 (45) | 248 (42) | 196 (47) | 282 (42) |
| *One larger bottle with several months in it* | 170 (15) | 44 (14) | 126 (16) | 28 (15) | 129 (16) | 13 (12) | 70 (14) | 100 (17) | 60 (14) | 110 (16) |
| *Unmarked (blank) container* | 78 (7) | 20 (6) | 58 (7) | 17 (9) | 60 (7) | 1 (1) | 38 (8) | 40 (7) | 29 (7) | 49 (7) |
| *Container with instruction label* | 78 (7) | 27 (9) | 51 (6) | 12 (6) | 59 (7) | 7 (6) | 42 (8) | 36 (6) | 34 (8) | 44 (6) |
| *Blister pack* | 147 (13) | 33 (11) | 114 (15) | 31 (16) | 104 (13) | 12 (11) | 61 (12) | 86 (15) | 50 (12) | 97 (14) |
| *Any kind of packaging is fine* | 143 (13) | 40 (13) | 103 (13) | 21 (11) | 101 (13) | 21 (19) | 62 (12) | 81 (14) | 48 (11) | 95 (14) |
| *Something else(specify)* | 4 (0) | 4 (1) | 0 (0) | 1 (1) | 2 (0) | 1 (1) | 3 (1) | 1 (0) | 2 (0) | 2 (0) |
| ***Preferred provider and service location*** |  |  |  |  |  |  |  |  |  |  |
| Preference for healthcare provider cadre |  |  |  |  |  |  |  |  |  |  |
| *Doctor or clinical officer* | 160 (15) | 64 (21) | 96 (12) | 17 (9) | 126 (16) | 17 (16) | 60 (12) | 100 (17) | 69 (16) | 91 (13) |
| *Nurse* | 872 (79) | 237 (76) | 635 (81) | 160 (85) | 625 (78) | 87 (80) | 421 (83) | 451 (76) | 325 (78) | 547 (81) |
| *Counsellor* | 52 (5) | 7 (2) | 45 (6) | 10 (5) | 38 (5) | 4 (4) | 19 (4) | 33 (6) | 21 (5) | 31 (5) |
| *CHW, peer/expert patient* | 7 (1) | 3 (1) | 4 (1) | 0 (0) | 7 (1) | 0 (0) | 4 (1) | 3 (1) | 4 (1) | 10 (1) |
| *Someone else (specify)* | 7 (1) | 1 (0) | 6 (1) | 1 (1) | 5 (1) | 1 (1) | 2 (0) | 5 (1) | 1 (0) | 6 (1) |
| Preferred service location |  |  |  |  |  |  |  | |  |  |
| *Community-based (e.g. at a school or church or pharmacy)* | 710 (65) | 206 (66) | 504 (64) | 113 (60) | 534 (67) | 63 (58) | 300 (59) | 410 (69) | 268 (64) | 442 (65) |
| *Home medication delivery* | 580 (53) | 168 (54) | 412 (52) | 94 (50) | 421 (53) | 65 (60) | 240 (47) | 340 (57) | 232 (55) | 348 (51) |
| ***Counselling and education preferences*** |  |  |  |  |  |  |  |  |  |  |
| Preference for quantity of one-on-one counselling to help manage your treatment, compared to what you received |  |  |  |  |  |  |  |  |  |  |
| *More* | 535 (49) | 153 (49) | 382 (49) | 89 (47) | 390 (49) | 56 (51) | 255 (50) | 280 (47) | 218 (52) | 317 (47) |
| *Same* | 540 (49) | 150 (48) | 390 (50) | 92 (49) | 396 (49) | 52 (48) | 242 (48) | 298 (50) | 191 (46) | 349 (51) |
| *Less* | 23 (2) | 9 (3) | 14 (2) | 7 (4) | 15 (2) | 1 (1) | 9 (2) | 14 (2) | 10 (2) | 13 (2) |
| Preference for quantity of information and education about HIV and ART, compared to what you received |  |  |  |  |  |  |  |  |  |  |
| *More* | 530 (48) | 150 (48) | 380 (48) | 89 (47) | 393 (49) | 48 (44) | 254 (50) | 276 (47) | 202 (48) | 328 (48) |
| *Same* | 538 (49) | 155 (50) | 383 (49) | 90 (48) | 390 (49) | 58 (53) | 246 (49) | 292 (49) | 205 (49) | 333 (49) |
| *Less* | 30 (3) | 7 (2) | 23 (3) | 9 (5) | 18 (2) | 3 (3) | 6 (1) | 24 (4) | 12 (3) | 18 (3) |
| Preference for format to receive information about HIV and ART** |  |  |  |  |  |  |  |  |  |  |
| *Written material (brochure or information sheet)* | 401 (37) | 129 (41) | 272 (35) | 59 (31) | 293 (37) | 49 (45) | 213 (42) | 188 (32) | 143 (34) | 258 (38) |
| *Class/group session in community (not at clinic)* | 89 (8) | 13 (4) | 76 (10) | 22 (12) | 57 (7) | 10 (9) | 23 (5) | 66 (11) | 45 (11) | 44 (6) |
| *Class/group session with provider at clinic* | 191 (17) | 51 (16) | 140 (18) | 20 (11) | 140 (17) | 31 (28) | 85 (17) | 106 (18) | 95 (23) | 96 (14) |
| *One-on-one session with provider at clinic* | 543 (49) | 165 (53) | 378 (48) | 93 (49) | 393 (49) | 57 (52) | 262 (52) | 281 (47) | 217 (52) | 326 (48) |
| *Social media (e.g. Facebook, Twitter)* | 241 (22) | 60 (19) | 181 (23) | 59 (31) | 171 (21) | 11 (10) | 68 (13) | 173 (29) | 99 (24) | 142 (21) |
| *Group within my community* | 43 (4) | 10 (3) | 33 (4) | 5 (3) | 32 (4) | 6 (6) | 16 (3) | 27 (5) | 22 (5) | 21 (3) |
| *Radio or TV* | 262 (24) | 76 (24) | 186 (24) | 33 (18) | 187 (23) | 42 (39) | 103 (20) | 159 (27) | 111 (26) | 151 (22) |
| *Videos to watch online at home* | 112 (10) | 28 (9) | 84 (11) | 25 (13) | 81 (10) | 6 (6) | 37 (7) | 75 (13) | 42 (10) | 70 (10) |
| *Text messages on my phone* | 589 (54) | 165 (53) | 424 (54) | 102 (54) | 435 (54) | 52 (48) | 293 (58) | 296 (50) | 215 (51) | 374 (55) |
| *Links to websites that I can browse in my own time* | 194 (18) | 47 (15) | 147 (19) | 50 (27) | 137 (17) | 7 (6) | 89 (18) | 105 (18) | 68 (16) | 126 (19) |
| *Other* | 3 (0) | 1 (0) | 2 (0) | 0 (0) | 3 (0) | 0 (0) | 1 (0) | 2 (0) | 2 (0) | 1 (0) |
| Preferred language for receiving information about HIV and ART |  |  |  |  |  |  |  |  |  |  |
| *English* | 422 (38) | 113 (36) | 309 (39) | 104 (55) | 297 (37) | 21 (19) | 147 (29) | 275 (46) | 166 (40) | 256 (38) |
| *isiZulu* | 301 (27) | 92 (29) | 209 (27) | 42 (22) | 218 (27) | 41 (38) | 202 (40) | 99 (17) | 79 (19) | 222 (33) |
| *Xitsonga* | 74 (7) | 26 (8) | 48 (6) | 9 (5) | 60 (7) | 5 (5) | 28 (6) | 46 (8) | 40 (10) | 34 (5) |
| *Siswati* | 165 (15) | 47 (15) | 118 (15) | 17 (9) | 129 (16) | 19 (17) | 87 (17) | 78 (13) | 82 (20) | 83 (12) |

*I/RI = Initiating ART on the day of PREFER study enrollment; $\leq$ 6 mos = On ART for 6 months or less at time of study enrollment

**Clients could select as many as apply

Supplementary Table 3. Participants’ preferences for treatment in the first six months by sex, age, and time on ART at enrolment, Zambia

| **Preference (n, %)** | **Total** | **Sex** | | | **Age groups in years** | | | **Place of residence** | | **Time on ART at study enrolment** | |
| --- | --- | --- | --- | --- | --- | --- | --- | --- | --- | --- | --- |
|  |  | **Male** | | **Female** | **18-24** | **25-49** | **50+** | **Rural** | **Urban** | **I/RI** | **≤6 months** |
| N | 771 | 257 | | 514 | 123 | 596 | 52 | 623 | 148 | 261 | 510 |
| ***Preferred visit and dispensing procedures*** |  |  | |  |  |  |  |  |  |  |  |
| Offered choice about service delivery since starting ART (yes) | 100 (13) | 30 (12) | | 70 (14) | 16 (13) | 79 (13) | 5 (10) | 83 (13) | 17 (11) | 31 (12) | 69 (14) |
| Preference for clinic visit frequency |  |  | |  |  |  |  |  |  |  |  |
| *Every month* | 61 (8) | 22 (9) | | 39 (8) | 11 (9) | 47 (8) | 3 (6) | 47 (8) | 14 (9) | 34 (13) | 27 (5) |
| *Every 2 months* | 72 (9) | 33 (13) | | 39 (8) | 14 (11) | 55 (9) | 3 (6) | 48 (8) | 24 (16) | 32 (12) | 40 (8) |
| *Every 3 months* | 233 (30) | 75 (29) | | 158 (31) | 34 (28) | 177 (30) | 22 (42) | 187 (30) | 46 (31) | 77 (30) | 156 (31) |
| *Every 6 months* | 372 (48) | 115 (45) | | 257 (50) | 56 (46) | 292 (49) | 24 (46) | 313 (50) | 59 (40) | 103 (39) | 269 (53) |
| *Other* | 33 (4) | 12 (5) | | 21 (4) | 8 (7) | 25 (4) | 0 (0) | 28 (4) | 5 (3) | 15 (6) | 18 (4) |
| Preferred part of the month to visit clinic |  |  | |  |  |  |  |  |  |  |  |
| *Early in the month (first week)* | 313 (41) | 99 (39) | | 214 (42) | 55 (45) | 238 (40) | 20 (38) | 242 (39) | 71 (48) | 105 (40) | 208 (41) |
| *Late in the month (last week)* | 187 (24) | 65 (25) | | 122 (24) | 28 (23) | 143 (24) | 16 (31) | 158 (25) | 29 (20) | 62 (24) | 125 (25) |
| *Middle of the month* | 96 (12) | 30 (12) | | 66 (13) | 18 (15) | 77 (13) | 1 (2) | 84 (13) | 12 (8) | 35 (13) | 61 (12) |
| *No preference* | 175 (23) | 63 (25) | | 112 (22) | 22 (18) | 138 (23) | 15 (29) | 139 (22) | 36 (24) | 59 (23) | 116 (23) |
| Preferred time(s) of day to come to clinic visits |  |  | |  |  |  |  |  |  |  |  |
| *Before work (before 8 am)* | 202 (26) | 72 (28) | | 130 (25) | 27 (22) | 158 (27) | 17 (33) | 146 (23) | 56 (38) | 70 (27) | 132 (26) |
| *Mornings (8 am to 12 pm)* | 429 (56) | 132 (51) | | 297 (58) | 67 (54) | 328 (55) | 34 (65) | 346 (56) | 83 (56) | 133 (51) | 296 (58) |
| *Lunch time (12 am to 2 pm)* | 41 (5) | 10 (4) | | 31 (6) | 6 (5) | 35 (6) | 0 (0) | 32 (5) | 9 (6) | 12 (5) | 29 (6) |
| *Afternoons (2 to 4 pm)* | 75 (10) | 33 (13) | | 42 (8) | 9 (7) | 63 (11) | 3 (6) | 64 (10) | 11 (7) | 29 (11) | 46 (9) |
| *After work (4-7 pm)* | 58 (8) | 21 (8) | | 37 (7) | 17 (14) | 40 (7) | 1 (2) | 53 (9) | 5 (3) | 26 (10) | 32 (6) |
| *Weekends* | 15 (2) | 5 (2) | | 10 (2) | 4 (3) | 11 (2) | 0 (0) | 15 (2) | 0 (0) | 7 (3) | 8 (2) |
| *Other* | 19 (2) | 5 (2) | | 14 (3) | 5 (4) | 14 (2) | 0 (0) | 19 (3) | 0 (0) | 6 (2) | 13 (3) |
| Preference for bringing companion (friend, family, support person) to clinic (yes) | 151 (20) | | 57 (22) | 94 (18) | 28 (23) | 114 (19) | 9 (17) | 114 (18) | 37 (25) | 63 (24) | 88 (17) |
| How many months of HIV medications did you receive today? |  | |  |  |  |  |  |  |  |  |  |
| *None* | 34 (4) | 11 (4) | | 23 (4) | 8 (7) | 24(4) | 2 (4) | 24 (4) | 10 (7) | 8 (3) | 26 (5) |
| *< One month* | 28 (4) | 12 (5) | | 16 (3) | 4 (3) | 22 (4) | 2 (4) | 23 (4) | 5(3) | 23 (9) | 5 (1) |
| *One month* | 274 (36) | 93 (36) | | 181 (35) | 52 (42) | 206 (35) | 16 (31) | 219 (35) | 55 (37) | 170 (65) | 104 (20) |
| *Two months* | 59 (8) | 23 (9) | | 36 (7) | 11 (9) | 43 (7) | 5 (10) | 38 (6) | 21 (14) | 6 (2) | 53 (10) |
| *Three months* | 334 (43) | 95 (37) | | 239 (47) | 42 (34) | 267 (45) | 25 (48) | 284 (46) | 50 (34) | 45 (17) | 289 (57) |
| *Four months* | 4 (0) | 3 (1) | | 1 (0) | 1 (1) | 3 (1) | 0 (0) | 2 (0) | 2 (1) | - | 4 (1) |
| *Six months* | 12 (2) | 6 (2) | | 6 (1) | 0 (0) | 11 (2) | 1 (2) | 10 (2) | 2 (1) | 2 (1) | 10 (2) |
| *Other* | 26 (3) | 14 (5) | | 12 (2) | 5 (4) | 20 (3) | 1 (2) | 23 (4) | 3 (2) | 7(3) | 19 (4) |
| Preference for how many months of medications to be dispensed |  |  | |  |  |  |  |  |  |  |  |
| *1 month at a time* | 39 (5) | 19 (7) | | 20 (4) | 12 (10) | 26 (4) | 1 (2) | 32 (5) | 7 (5) | 26 (10) | 13 (3) |
| *2 months at a time* | 59 (8) | 29 (11) | | 30 (6) | 9 (7) | 48 (8) | 2 (4) | 44 (7) | 15 (10) | 26 (10) | 33 (6) |
| *3 months at a time* | 237 (31) | 78 (30) | | 159 (31) | 30 (24) | 185 (31) | 22 (42) | 194 (31) | 43 (29) | 81 (31) | 156 (31) |
| *4 months at a time* | 17 (2) | 7 (3) | | 10 (2) | 5 (4) | 11 (2) | 1 (2) | 12 (2) | 5 (3) | 6 (2) | 11 (2) |
| *6 months at a time* | 419 (54) | 124 (48) | | 295 (57) | 67 (54) | 326 (55) | 26 (50) | 341 (55) | 78 (53) | 122 (47) | 297 (58) |
| Preference for medication packaging |  |  | |  |  |  |  |  |  |  |  |
| *One bottle for each month* | 270 (35) | 93 (36) | | 177 (34) | 32 (26) | 219 (37) | 19 (37) | 206 (33) | 64 (43) | 87 (33) | 183 (36) |
| *One larger bottle with several months in it* | 277 (36) | 85 (33) | | 192 (37) | 44 (36) | 210 (35) | 23 (44) | 227 (36) | 50 (34) | 79 (30) | 198 (39) |
| *Unmarked (blank) container* | 53 (7) | 12 (5) | | 41 (8) | 21 (17) | 30 (5) | 2 (4) | 45 (7) | 8 (5) | 21 (8) | 32 (6) |
| *Container with instruction label* | 84 (11) | 31 (12) | | 53 (10) | 12 (10) | 71 (12) | 1 (2) | 68 (11) | 16 (11) | 38 (15) | 46 (9) |
| *Blister pack* | 41 (5) | 17 (7) | | 24 (5) | 8 (7) | 30 (5) | 3 (6) | 38 (6) | 3 (2) | 23 (9) | 18 (4) |
| *Any kind of packaging is fine* | 41 (5) | 17 (7) | | 24 (5) | 3 (2) | 34 (6) | 4 (8) | 34 (5) | 7 (5) | 12 (5) | 29 (6) |
| *Something else(specify)* | 5 (1) | 2 (1) | | 3 (1) | 3 (2) | 2 (0) | 0 (0) | 5 (1) | 0 (0) | 1 (0) | 4 (1) |
| ***Preferred provider and service location*** |  |  | |  |  |  |  |  |  |  |  |
| Preference for healthcare provider |  |  | |  |  |  |  |  |  |  |  |
| *Doctor or clinical officer* | 382 (50) | 148 (58) | | 234 (46) | 63 (51) | 292 (49) | 27 (52) | 308 (49) | 74 (50) | 122 (47) | 260 (51) |
| *Nurse* | 200 (26) | 55 (21) | | 145 (28) | 30 (24) | 154 (26) | 16 (31) | 166 (27) | 34 (23) | 62 (24) | 138 (27) |
| *Counsellor* | 158 (20) | 43 (17) | | 115 (22) | 25 (20) | 126 (21) | 7 (13) | 120 (19) | 38 (26) | 71 (27) | 87 (17) |
| *CHW, peer/expert patient* | 14 (2) | 4 (2) | | 10 (2) | 4 (3) | 9 (2) | 1 (2) | 13 (2) | 1 (1) | 2 (1) | 12 (2) |
| *Someone else (specify)* | 17 (2) | 7 (3) | | 10 (2) | 1 (1) | 15 (3) | 1 (2) | 16 (3) | 1 (1) | 4 (2) | 13 (3) |
| Preference for service delivery location |  |  | |  |  |  |  |  |  |  |  |
| *Community-based (e.g. at a school or church or pharmacy)* | 232 (30) | 77 (30) | | 155 (30) | 30 (24) | 196 (33) | 6 (12) | 196 (31) | 36 (24) | 76 (29) | 156 (31) |
| *Home medication delivery* | 439 (57) | 151 (59) | | 288 (56) | 64 (52) | 350 (59) | 25 (48) | 344 (55) | 95 (64) | 155 (59) | 284 (56) |
| ***Counselling and education preferences*** |  |  | |  |  |  |  |  |  |  |  |
| Preference for quantity of one-on-one counselling to help manage your treatment, compared to what you received |  |  | |  |  |  |  |  |  |  |  |
| *More* | 372 (48) | 118 (46) | | 254 (49) | 68 (55) | 279 (47) | 25 (48) | 290 (47) | 82 (55) | 129 (49) | 243 (48) |
| *Same* | 369 (48) | 131 (51) | | 238 (46) | 50 (41) | 294 (49) | 25 (48) | 307 (49) | 62 (42) | 124 (48) | 245 (48) |
| *Less* | 30 (4) | 8 (3) | | 22 (4) | 5 (4) | 23 (4) | 2 (4) | 26 (4) | 4 (3) | 8 (3) | 22 (4) |
| Preference for quantity of information and education about HIV and ART, compared to what you received |  |  | |  |  |  |  |  |  |  |  |
| *More* | 372 (48) | 114 (44) | | 258 (50) | 72 (59) | 276 (46) | 24 (46) | 300 (48) | 72 (49) | 130 (50) | 242 (47) |
| *The same* | 369 (48) | 136 (53) | | 233 (45) | 45 (37) | 298 (50) | 26 (50) | 300 (48) | 69 (47) | 122 (47) | 247 (48) |
| *Less* | 30 (4) | 7 (3) | | 23 (4) | 6 (5) | 22 (4) | 2 (4) | 23 (4) | 7 (5) | 9 (3) | 21 (4) |
| Preference for format to receive information about HIV and ART** |  |  | |  |  |  |  |  |  |  |  |
| *Written material (brochure or information sheet)* | 171 (22) | 61 (24) | | 110 (21) | 33 (27) | 123 (21) | 15 (29) | 137 (22) | 34 (23) | 67 (26) | 104 (20) |
| *Class/group session in community (not at clinic)* | 51 (7) | 15 (6) | | 36 (7) | 10 (8) | 36 (6) | 5 (10) | 38 (6) | 13 (9) | 20 (8) | 31 (6) |
| *Class/group session with provider at clinic* | 131 (17) | 34 (13) | | 97 (19) | 29 (24) | 95 (16) | 7 (13) | 112 (18) | 19 (13) | 35 (13) | 96 (19) |
| *One-on-one session with provider at clinic* | 350 (45) | 116 (45) | | 234 (46) | 56 (46) | 271 (45) | 23 (44) | 258 (41) | 92 (62) | 114 (44) | 236 (46) |
| *Social media (e.g. Facebook, Twitter)* | 55 (7) | 24 (9) | | 31 (6) | 9 (7) | 44 (7) | 2 (4) | 45 (7) | 10 (7) | 21 (8) | 34 (7) |
| *Group within my community* | 19 (2) | 5 (2) | | 14 (3) | 5 (4) | 13 (2) | 1 (2) | 14 (2) | 5 (3) | 8 (3) | 11 (2) |
| *Radio or TV* | 183 (24) | 63 (25) | | 120 (23) | 26 (21) | 139 (23) | 18 (35) | 146 (23) | 37 (25) | 63 (24) | 120 (24) |
| *Videos to watch online at home* | 13 (2) | 4 (2) | | 9 (2) | 2 (2) | 11 (2) | 0 (0) | 13 (2) | 0 (0) | 2 (1) | 11 (2) |
| *Text messages on my phone* | 236 (31) | 93 (36) | | 143 (28) | 34 (28) | 191 (32) | 11 (21) | 185 (30) | 51 (34) | 84 (32) | 152 (30) |
| *Links to websites that I can browse in my own time* | 6 (1) | 2 (1) | | 4 (1) | 2 (2) | 3 (1) | 1 (2) | 5 (1) | 1 (1) | 2 (1) | 4 (1) |
| *Other* | 4 (1) | 1 (0) | | 3 (1) | 0 (0) | 4 (1) | 0 (0) | 4 (1) | 0 (0) | 3 (1) | 1 (0) |
| Preferred language for receiving information about HIV and ART |  |  | |  |  |  |  |  |  |  |  |
| *English* | 128 (17) | 48 (19) | | 80 (16) | 29 (24) | 89 (15) | 10 (19) | 114 (18) | 14 (9) | 41 (16) | 87 (17) |
| *Nyanja* | 346 (45) | 109 (42) | | 237 (46) | 56 (46) | 274 (46) | 16 (31) | 262 (42) | 84 (57) | 107 (41) | 239 (47) |
| *Bemba* | 217 (28) | 71 (28) | | 146 (28) | 27 (22) | 172 (29) | 18 (35) | 205 (33) | 12 (8) | 84 (32) | 133 (26) |
| *Tonga* | 55 (7) | 20 (8) | | 35 (7) | 8 (7) | 42 (7) | 5 (10) | 26 (4) | 29 (20) | 20 (8) | 35 (7) |

*I/RI = Initiating ART on the day of PREFER study enrollment; $\leq$ 6 mos = On ART for 6 months or less at time of study enrollment

**Clients could select as many as apply

File S1. Survey instrument
