# Supplementary material for: Experiences and Preferences in Zambia and South Africa for Delivery of HIV Treatment During a Client’s First Six Months: Results of the PREFER Study’s Cross-Sectional Baseline Survey
Source: AIDS Behav. 2025 Feb 1;29(6):1713–28. doi: 10.1007/s10461-025-04640-y (PMC12075270; doi:10.1007/s10461-025-04640-y)
Supplement: Supplementary file 2 — Supplementary file1 (PDF 51 kb) [file 10461_2025_4640_MOESM2_ESM.pdf]

Screening number

PART A: SCREENING

Surveyor ID ____________________________________ Facility Name ____________________________

Date (DD/MM/YYYY) ____________________________

I. Introduction

This survey should be administered to patients who have been on ART for less than 6 months during their current treatment period, including those initiating ART today. If the patient is initiating today, this questionnaire must be administered AFTER the patient completes their regular clinic visit. If the patient initiated before today, this questionnaire can be administered BEFORE the regular clinic visit, while the patient is waiting in the queue.

Ask the patient for a few minutes of their time. Introduce yourself and the study. Then please read the following statement to the patient:

> *"You are being asked to voluntarily give us some information to see if you might qualify to be enrolled in a research study. We are asking you to be in this study because you recently started taking treatment for HIV (ART) at this facility, and we are doing research on how to make it easier for new ART patients to stay in care during their first few months after starting. If you agree, we will ask you to tell us your age, gender, and how long ago you started ART. This information will tell us if you are qualified to enrol in the study.*
>
> *If you qualify for the study and decide to join, the information that we get from you will become part of your study record. All study procedures will be conducted in a confidential space and that the information collected or shared during the conduct of research study activities will not be available to those outside of the study team. If you do not qualify or decide not to join the study, the information that we get from you will only be used to keep track of how many patients we have invited into the study and if those who do qualify and decide to join are different in age, sex, or time on ART from those who do not join. We will keep this information until we have finished enrollment for the study, then destroy it.*
>
> *Saying yes to this screening does not mean you have to agree to be in the study. If you have any questions, please ask them now or at any time you can contact Dr. Mhairi Maskew, Principal Researcher, Health Economics and Epidemiology Research Office, Johannesburg, telephone 010 001 7930 or email mmaskew@heroza.org."*

If the patient agrees to be screened, give the patient the information sheet for the study. If the patient does not wish to be screened, thank them for their time and end the interaction without collecting any information.

If the patient agrees to be screened, proceed to section II below and then complete the eligibility screening in section III.

II. Demographic description

1. Gender: Male____ Female ____ Other/unknown ____

2. How old were you at your last birthday? _________ (years)

III. Eligibility screening

3. Are you starting ART today?

   YES ________ (Skip next question)

   NO ________ (Proceed with next question)

4. If you are not starting today, when did you most recently start ART (month/year)?

Interviewer: if patient does not recall when he or she started but is carrying a file or ART patient card, you can ask to look at it to determine the month and year ART started.

Patient started ART >6 months ago ___ (STOP! Thank the participant for their time but do not proceed with the survey)
Patient started ART <6 months ago ___ (Proceed with next question)

5. Are you currently age 18 or older?

YES ________ (Proceed with next question)

NO ________ (STOP! Thank the participant for their time but do not proceed with the survey)

IV. Eligibility decision

This patient is

ELIGIBLE for the study _______ (Please proceed with the informed consent process)

NOT ELIGIBLE for the study_______(Thank the participant for their time but do not proceed with the survey)

This patient

CONSENTED TO PARTICIPATE____ (Fill in survey instrument ID number below and proceed with survey)

DID NOT CONSENT TO PARTICIPATE ____ (Thank the participant for their time and end the interaction)

If the participant does not consent to being in the study, thank them for their time and end the interaction.

PART B: SURVEY

Survey ID

Location of interview within facility:

Interviewer notes:

Introduction

Interviewer: Read the following statement. Please repeat the statement translated into the local language based on primary languages.

"Thank you for agreeing to participate in this survey. My name is ______. I will be asking you the questions. Most of the questions require that you select one of the options as your answer, although some questions you can select all the answers that apply. I will specify the options and instructions for you as I ask each question. If your answer is not one of the specified options please tell me and I will write your answer down.

Please feel free to tell me whatever you are comfortable sharing. You should also remember that you do not have to share anything that you are not comfortable sharing and that you can stop this interview at any time without any risk to your rights or treatment and care. There are no right or wrong answers, so please be honest and help us to understand what is true for you and your community. Are you ready to begin?

I'm going to start by asking you some basic questions about who you are, where you live, and your education and employment."

Note: Skip patterns will be programmed into the electronic data collection form

Q#	QUESTION	RESPONSES
**I. Respondent demographics and socio-economic status**		
1.	Patient file/ART number	Record from file
2.	What are your telephone (mobile) number and e-mail address, in case we would like to contact you after today?	Record answer; collect both if available, otherwise either one
3.	What is your relationship status?	1= I have a primary partner or spouse who I live with<br>2= I have a primary partner or spouse but we do not live together<br>3= I do not have a primary partner or spouse at this time<br>4= Other
4.	Do you think of the house you currently live in as your main house?	1= Yes<br>2= No, my main house is somewhere else in South Africa<br>3= No, my main house is in another country
5.	How long have you lived in your current neighbourhood/community?	Months/years
6.	What is your reading level?	1= Read well<br>2= Read somewhat<br>3= Cannot read
7.	How comfortable are you speaking English?	1= I am very comfortable<br>2= I am somewhat comfortable<br>3= I am not comfortable
8.	How comfortable are you using a mobile phone or computer for receiving information?	1= I am very comfortable<br>2= I am somewhat comfortable<br>3= I am not comfortable
9.	What was the highest level of school that you completed?	1= No schooling<br>2= Primary<br>3= Secondary<br>4= Certificate/Diploma/Post-secondary<br>5= Graduate degree

Q#	QUESTION	RESPONSES
10.	What is your primary occupation?	1= Formal sector job (salaried)<br>2= Informal sector job (not farming or domestic) (e.g. trader, day service provider)<br>3= Farming (my own or my family's farm)<br>4= Farm worker (someone else's farm)<br>5= Domestic worker or carer (paid)<br>6= Household work and/or childcare (my own house, not paid)<br>7= Unemployed, looking for work<br>8= Unemployed, not looking for work<br>9= Student or trainee<br>10= Retired<br>11= Other (specify)
11.	What time of day do you usually work?	1= All day (regular working day)<br>2= Mornings only<br>3= Afternoons only<br>4= Nights only<br>5= Weekends<br>6= Flexible times (work for myself)<br>7= Shifts change from day to day<br>8= I do not work
12.	How many living children do you have?	Specify number (0 if no children)
13.	How many adults and children are living in your household, including yourself? By "living in," I mean they spend most nights of the week sleeping in your household.	Specify number
14.	How many other people in your household have HIV, to your knowledge?	Specify number
15.	How many other people in your household are on ART, to your knowledge?	Specify number
16.	Do you have electricity in your house?	0= No<br>1= Yes
17.	Do you have access to piped water?	1= No<br>2= Yes – to house<br>3= Yes – community tap/pipe
18.	Do you or the people in your household go without food often, sometimes, seldom, never?	1= Never<br>2= Seldom<br>3= Sometimes<br>4= Often
19.	If a person in your household became ill and 100 Rands was needed for treatment or medicines, would you say it would be very easy, easy, difficult, or very difficult to find the money?	1= Very difficult<br>2= Difficult<br>3= Easy<br>4= Very easy

II. HIV testing history

Now we would like to ask you about your experience in testing for HIV and taking HIV treatment (antiretroviral medications). We will use the term "current treatment" to refer to the period from when you most recently started taking ARVs until today. If you are just starting treatment today, then your "current treatment" periods start today. If you took ARVs at any time before and then stopped taking treatment for at least 3 months, before re-starting this time, we will call that your "earlier treatment" period. If you took ARVs more than once in the past and stopped taking treatment more than once, the "earlier treatment" period will be your most recent time on ARVs, before this one.

Q#	QUESTION	RESPONSES
20.	Have you ever been tested positive for HIV before you started ART this time (current treatment)?	0= No<br>1= Yes<br>2= Don't know/can't remember
21.	If you've ever tested positive for HIV before the current treatment period, when was that test?	1= <3 months ago<br>2= <6 months ago<br>3= <12 months ago<br>4= > 1 year ago<br>5= >5 years ago<br>6= I never had a positive HIV test prior to the current treatment period

Q#	QUESTION	RESPONSES
22.	If you've ever tested positive for HIV before the current treatment period, where did you test?	1= Public clinic or hospital<br>2= Private provider<br>3= Mobile HIV testing van<br>4= At home (self-test)<br>5= At home (by a healthcare worker who came to your home)<br>6= Other (specify)
23.	Thinking about your <u>first</u> positive HIV test, which could have been today or in the past, do you recall why you got tested at the time?	1= Testing recommended by healthcare provider<br>2= Known exposure or risk<br>3= Ill health<br>4= Employment or medical aid purposes<br>5= Tested as infant or child<br>6= Other (specify)
24.	How long did it take from the time you got tested to be initiated on HIV treatment <u>the first time you started HIV treatment</u>? (Note: this pertains to the respondent's first time on ART, even if that is not the most recent time)	1= Same day as diagnosis<br>2= 2-3 days<br>3= Week<br>4= Month<br>5= Longer than a month<br>6= Longer than 6 months<br>7= Longer than 1 year<br>8= Don't know/can't remember
25.	Thinking about your <u>most recent</u> HIV test, how did you find the testing experience overall? (Choose all that apply)	1= It encouraged me to start treatment<br>2= It encouraged me to seek more information or take other steps to improve my health<br>3= It did not support me to start treatment<br>4= It made me less likely to start treatment<br>5= I found it to be supportive or empowering<br>6= I found it to be discouraging or frightening<br>7= It did not affect how I felt about treatment<br>8= Don't know/can't remember
**III.**	**HIV treatment history**	
26.	For how long have you been taking ARVs as HIV treatment during your current treatment period (this one)?	0= Starting today<br>____Months (must be ≤6)
27.	Did you ever take treatment previously, either here or at any other healthcare facility, and then stop for at least 3 months (did you ever have an earlier treatment period)?	Yes/no
28.	How many times did you take treatment previously, with stops for at least 3 months between them?	Number; answer must be ≥1
29.	When did you stop taking treatment previously?	Year/month
30.	For how long were you on treatment previously, before you stopped?	Years/months
31.	What were the most important reasons for your stopping treatment previously? You can list up to 3 reasons, in order of importance.	1= I felt healthy and did not think I still needed treatment<br>2= I did not like the side effects<br>3= I was worried about stigma/other people's opinions if they learned I was on ART<br>4= I was afraid I'd lose my job<br>5= I ran out of medications<br>6= I did not have time to come to the clinic for visits or to pick up medication<br>7= I could not afford to come to the clinic for visits or to pick up medication<br>8= The clinic was too unfriendly; I did not want to come back<br>9= I relocated to another area<br>10= I could not collect my medication because of COVID-19 restrictions<br>11= Other (specify)

Q#	QUESTION	RESPONSES
32.	Did you ever in the past take ARVs for reasons other than as treatment for your own HIV infection (e.g. as PrEP, PEP, or PMTCT)?	Yes/no
33.	Since the first time you had a positive HIV test, have you ever had any health problems, symptoms, or illnesses that you believe were caused by having HIV?	Yes/no
34.	If yes, please describe	Open ended
35.	At the time you most recently started ART (current treatment period), how did you feel physically?	1= I felt very sick<br>2= I felt a little sick<br>3= I felt fine, not sick at all
36.	How do you feel physically today? (Only for participants who started ART before today)	1= I feel very sick<br>2= I feel a little sick<br>3= I feel fine, not sick at all
**IV.**	**Current HIV care and treatment experience**	
37.	What is the main reason for you coming to this facility today?	1= HIV test<br>2= Scheduled HIV treatment initiation following positive HIV test<br>3= Regular visit 1 month after initiation<br>4= Regular visit 2 months after initiation<br>5= Regular visit 3 months after initiation<br>6= Medication refill only<br>7= Unscheduled visit related to HIV or ART<br>8= Not for HIV—referred here from elsewhere in the clinic<br>9= Other (specify)
38.	How many months of HIV medications did you receive today?	1= None<br>2= 1 month<br>3= 2 months<br>4= 3 months<br>5= Other (specify)
39.	Did you receive counselling before starting ART? (Counseling about treatment, not HIV test counseling)	0= No<br>1= Yes
40.	Did the counselling you received make you feel better/the same/worse about starting ART?	1= Better<br>2= The same<br>3= Worse
41.	After counselling, how did you feel about starting treatment for HIV?	1= Encouraged to start treatment<br>2= Motivated to seek more information or take other steps to improve my health<br>3= I found it to be supportive or empowering<br>4= It did not affect how I felt about treatment<br>5= It did not support me to start treatment<br>6= It made me less likely to start treatment<br>7= I found it to be discouraging or frightening<br>8= Don't know/can't remember
42.	If you had a question about your HIV treatment, what would you most likely do?	1= Make an unscheduled visit to this clinic<br>2= Wait and ask during a regular visit to this clinic<br>3= Wait and ask during a regular out-of-facility HIV event<br>4= Ask a friend or family member who is also positive<br>5= Search on the internet/library<br>6= Other (specify)
43.	Since initiating ART, have you experienced any health problems, symptoms, or illness that you thought was a side effect of the treatment? (Note: question only for those who started ART before today)	Yes/no
44.	If yes, please describe the side effects	Open ended
45.	If yes, what did you do about the side effects?	1= Nothing, took no action

Q#	QUESTION	RESPONSES
		2= Cared for myself at home (e.g. pain relievers, etc.)<br>3= Came back to this clinic for an unscheduled visit<br>4= Went to a hospital or other urgent care facility<br>5= Waited for my scheduled visit, then asked the healthcare worker what to do<br>6= Went to another provider (specify)<br>7= Other (specify)
46.	Have there been occasions where you missed scheduled facility visits in the past year by more than 2-3 days?	0= No<br>1= Yes<br>2= Don't know/can't remember
47.	How many visits have you missed?	Specify number
48.	Why did you miss the visit?	1= Forgot pick up date<br>2= Ill health<br>3= Nobody else to go for me<br>4= Nobody else to come with me (buddy)<br>5= Clinic not open at times I could go<br>6= Waiting times were too long<br>7= No money for transport<br>8= Could not leave work<br>9= Afraid HIV status will get known<br>10= Traveling or away from home at that time<br>11= Other (specify)
49.	Did you come alone to the clinic today or with someone else? (Choose all that apply)	1= Alone<br>2= With one or more of my children<br>3= With someone else in my family<br>4= With a friend or co-worker<br>5= With another patient<br>6= Other (specify)
50.	What difficulties do you expect to have in taking your medications every day?<br>a. I might forget to take my medication<br>b. I may feel side effects<br>c. No food to eat when taking medication<br>d. No privacy to take ART without others seeing<br>e. medication before I'm able to get to the clinic<br>f. The hours I work make it difficult to take medication<br>g. I may not have time to come to the clinic for visits or to pick up medication<br>h. I could not afford to come to the clinic for visits or to pick up medication<br>i. The clinic was too unfriendly; I did not want to come back<br>j. I relocated to another area<br>k. Collecting my medication because of COVID-19 restrictions<br>l. Other (specify)	For each of these, select one of the options:<br>0= No difficulties<br>1= Some or moderate difficulties<br>2= A lot of difficulties
51.	Do you expect to have any difficulties in picking up your medications every month?	1= Yes<br>2= No<br>3= Maybe
52.	If yes or maybe, what difficulties do you expect?	Open ended
53.	Since your first positive HIV test, about how many people have you told that you have HIV?	Number (0 for not disclosed to anyone)
54.	At this time, about how many other people do you think know that you have HIV, including people you've told and people who may have learned it from someone else.	1= No one<br>2= Just 1 or 2 others<br>3= 3-5 others<br>4= 6-10 others<br>5= More than 10 others/lots of people know<br>6= Don't know
55.	Does at least one other person living in your household know you have HIV?	1= Yes<br>2= No

Q#	QUESTION	RESPONSES
		3= Live alone
56.	Does your partner or husband or wife know you have HIV?	1= Yes<br>2= No<br>3= No partner
57.	Do you know anyone who has been taking ART for at least one year and is doing well?	Yes/no
**V.**	**Other healthcare**	
58.	What chronic (long-term) diseases, conditions, or healthcare needs do you currently have in addition to HIV, if any? (Tick all that apply)	1= No other diseases or healthcare needs<br>2= TB<br>3= Diabetes<br>4= Hypertension<br>5= Asthma<br>6= Mental health concerns<br>7= Contraception/family planning<br>8= Nutritional/dietary assistance<br>9= Pain management (chronic pain)<br>10= Pregnancy/post-partum care<br>11= Other (specify)
59.	If you have any other diseases, conditions, or healthcare needs, do you come to this clinic for services?	Yes/no
60.	If you have any other diseases, conditions, or healthcare needs, how often do you come to this clinic for services for the other diseases, conditions, or healthcare needs you mentioned?	1= Monthly<br>2= Every 2 months<br>3= Every 3 months<br>4= Every 6 months<br>5= Annually<br>6= Other (specify)
61.	In the past 12 months have you sought health care from any other health care provider outside this facility? (Tick all that apply)	1= No, no care from another provider<br>2= Other public clinic<br>3= Hospital<br>4= Private doctor<br>5= Traditional healer/sangoma<br>6= Community health worker<br>7= Local community group/NGO/FBO<br>8= Other (specify)
62.	If yes, for what purpose did you seek services outside this facility?	Open ended
63.	Have you received ARV medications from anyone else outside this clinic (another clinic, private facility, etc.) in the past 3 months?	Yes/no
64.	If yes, from whom did you receive ARV medications outside this clinic?	1= Another public clinic or hospital<br>2= Private GP or other provider<br>3= Private pharmacy<br>4= CCMDD<br>5= Shared by a friend or family member<br>6= Somewhere else (specify)
65.	Have you ever taken any non-ARV medications or tried anything else to manage your HIV?	Yes/no
66.	If yes, what non-ARV medications or other things have you tried? Please be specific.	Open ended
67.	When you started HIV treatment most recently, were you asked about a cough, fever, weight loss or sweating at night?	1= Yes<br>2= No<br>3= Don't remember
68.	Did you have a TB test when you started HIV treatment most recently?	Yes/no/don't remember
69.	If yes, were you told that you should wait to start HIV treatment until you received your TB test result or started TB treatment?	Yes/no/don't remember
70.	Have you ever been diagnosed with TB (tuberculosis)?	Yes/no
71.	If yes, when did you receive this diagnosis?	1= Today<br>2= In the past 1 month<br>3= In the past year

Q#	QUESTION	RESPONSES
		4= More than 1 year ago
72.	Have you ever been treated for TB before?	Yes/no
73.	If yes, how many times have you been treated for TB before?	Number
74.	If yes, when did you start your most recent TB treatment?	1= Today<br>2= In the past 1 month<br>3= In the 6 months<br>4= More than 6 months ago
75.	If you started TB treatment more than 6 months ago, did you complete TB treatment?	1= Yes<br>2= No<br>3= Unsure
76.	If you started TB treatment within the past 6 months, did you start TB treatment after or before you started HIV treatment?	1= I started TB treatment before HIV treatment<br>2= I started TB treatment after HIV treatment<br>3= I started both at the same time<br>4= Don't know/can't remember
**VI.**	**Patient preferences**	
77.	Since you started ART, have you been offered any choices about where, when, or how often you should pick up your medications? (For example, were you offered any choice about when your next clinic visit should be, or how many months of medication you would like at once?)	1= Yes<br>2= No<br>3= Don't know
78.	If yes, what choice(s) were you offered? Please be specific.	Open ended
79.	If you had a choice, how often would you like to come to this clinic for HIV care?	1= Every month<br>2= Every 2 months<br>3= Every 3 months<br>4= Every 6 months<br>5= Other (specify)
80.	If you had a choice, how many months of medications would you like to be given at a time?	1= 1 month at a time<br>2= 2 months at a time<br>3= 3 months at a time<br>4= 4 months at a time<br>5= 6 months at a time
81.	If you had a choice, what part of the month would you prefer to come to the clinic?	1= Early in the month (first week)<br>2= Late in the month (last week)<br>3= Middle of the month<br>4= Doesn't matter, can come any time during the month
82.	What day(s) of the week are most convenient for you to come to clinic visits? (check all that apply)	1= Monday<br>2= Tuesday<br>3= Wednesday<br>4= Thursday<br>5= Friday<br>6= Saturday<br>7= Sunday
83.	What time(s) of the day are most convenient for you to come to clinic visits (select all that apply)	1= Before work in the morning (before 8 am)<br>2= Mornings (8 am to 12 pm)<br>3= Lunch time (12 am to 2 pm)<br>4= Afternoons (2 to 4 pm)<br>5= After work in the early evening (4-7 pm)<br>6= Weekends<br>7= Other (specify)
84.	Do you prefer to come to the clinic alone or with someone else?	1= Alone<br>2= With a family member<br>3= With a friend<br>4= With someone else (specify)<br>5= Doesn't matter/no preference
85.	If you could receive HIV treatment somewhere outside the clinic (e.g. at a school or church or pharmacy), would you prefer that to coming to the clinic?	Yes/no

Q#	QUESTION	RESPONSES
86.	If yes, where would be the best place for you to receive treatment? (Be specific)	Open ended
87.	If you had the chance for your medications to be delivered to you at home, either by a community health worker or by a courier, would you prefer that?	Yes/no
88.	Where do you or will you store your medication? (Be specific, e.g. bathroom cabinet, my bedroom, in my bag, in my desk at work, etc.)	Open ended
89.	If you had a choice, how would you like your medications to be packaged?<br>1= One bottle for each month<br>2= One larger bottle with several months in it<br>3= An unmarked (blank) container<br>4= A container with instructions on it<br>5= A blister pack<br>6= Any kind of packaging is fine<br>7= Something else(specify)<br>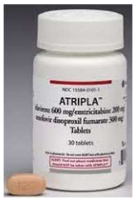 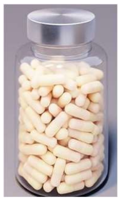 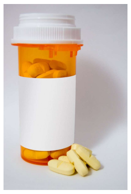 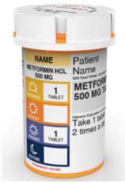 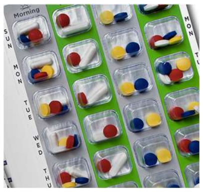 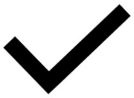 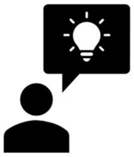	Show pictures with choices
90.	What kind of healthcare provider would you most like to see when receive HIV services after today?	1= Doctor or clinical officer<br>2= Nurse<br>3= Counsellor<br>4= Community health worker<br>5= Peer/expert patient<br>6= Someone else (specify)
91.	Do you wish that this clinic offered you more/the same/less information and education about HIV and ART?	1= More<br>2= The same<br>3= Less
92.	Are there any topics or types of information you wish you'd learned more about from this clinic?	Open ended
93.	Do you wish that this clinic offered you more/the same/less one-on-one counselling (with a counsellor) to help you manage your HIV and treatment?	1= More<br>2= The same<br>3= Less
94.	In what format would you most like to receive information about HIV and ART? (Choose up to 3, in order of preference)	1= Written material (brochure or information sheet) that I can take away<br>2= Class/group session in community (not at clinic)<br>3= Class/group session with provider at clinic<br>4= One-on-one session with provider at clinic<br>5= Social media (e.g. Facebook, Twitter)<br>6= Community group in my community<br>7= Radio or TV<br>8= Videos I can watch online at home<br>9= Text messages on my phone<br>10= Links to websites that I can browse in my own time<br>11= Other (specify)
95.	In what language are you most comfortable receiving information about HIV and ART?	1= English<br>2= isiZulu<br>3= isiXhosa<br>4= Setswana<br>5= Xitsonga<br>6= isiNdebele<br>7= Sesotho

Q#	QUESTION	RESPONSES
		8= Siswati<br>9= Tshivenda<br>10= Sepedi<br>11= Afrikaans<br>12= Other (specify)
96.	If you could change any one thing about how you receive care, what would it be?	Open ended
**VII.**	**Expectations of care**	
97.	When you visit the clinic, about how long do you expect the visit to last, from when you walk in until when you leave?	Hours/minutes
98.	When you visit the clinic, how long do you expect to wait in a queue before you receive service?	Hours/minutes
99.	Is the care you receive at this clinic as good as/better than/worse than you expect it to be?	1= As good as<br>2= Better than<br>3= Worse than
100.	Please state how much you agree or disagree with the following statements<br><br>a. The doctors and nurses discussed the treatment fully with me<br>b. I find it easy to tell the heath workers when I have missed taking my tablets<br>c. It is a problem that health workers do not speak my language<br>d. The health workers are too busy to listen to my problems<br>e. Some staff do not treat patients with sufficient respect<br>f. The facilities are dirty<br>g. The queues to see a doctor or nurse are too long at this facility<br>h. In this clinic you're able to talk to the doctors or nurses in private	These are all Likert scales 1-5 (strongly disagree, mildly disagree, neither agree or nor disagree, mildly agree, strongly agree, no response/don't know)
101.	What do you find most frustrating about getting care at this clinic?	Open ended
102.	What is the one thing that the clinic could do to make it easier for you to stay on ART for the coming year?	Open ended
**VIII.**	**Costs of seeking care**	
103.	How do you usually get to the clinic? (Tick all that apply)	1= Walk<br>2= Mini-bus/common taxi<br>3= Own car<br>4= Meter taxi/Uber/Bolt<br>5= Brought by family/friends in their vehicles<br>6= Other (specify)
104.	How long does it take you to get to the clinic? (One way – from home to the clinic)	Hours/minutes one way
105.	What kinds expenses/costs do you incur for each clinic visit? (Return trip, from when you leave home until you return home) (Tick all that apply)	0= No costs<br>1= Transport<br>2= Loss of income due to missing work<br>3= Child care<br>4= Food/drinks<br>5= Other (specify)
106.	Please estimate how much transport costs you in Rands each time you visit the clinic, including taxi or bus fare, petrol for a car, etc. (Return trip – to the clinic and back home)	Amount in Rands
107.	Do you have to miss or take time off work in order to visit the clinic?	Yes/no
108.	If yes, how much work time do you miss or take off for each clinic visit, on average?	1= <1 hour<br>2= 1-2 hours<br>3= 2-3 hours<br>4= ½ day<br>5= Full day

Q#	QUESTION	RESPONSES
109.	How much does unpaid time off work cost you in Rands each time you visit the clinic?	Amount in Rands
**IX.**	**Conclusion of interview**	
110.	Is there anything else you'd like me to know about the care you receive, your concerns about treatment, your preferences for how you receive treatment, or any of the other topics we've talked about today?	Open ended

Surveyor: Please thank the participant for their time and ask if they have any additional questions about the study.

PART C: WRAPUP

Surveyor initials ___________

Supervisor initials ___________

Date reviewed by supervisor ___________

Comments/notes to team:
